# Transcriptional regulation of disease-relevant microglial activation programs

**DOI:** 10.1101/2025.10.12.681832

**Published:** 2025-10-27

**Authors:** Amanda McQuade, Reet Mishra, Venus Hagan, Weiwei Liang, Peter J. Colias, Vincent Cele Castillo, Justin P. Lubin, Verena Haage, Victoria Marshe, Masashi Fujita, Layla Gomes, Thomas Ta, Olivia Teter, Sarah E. Chasins, Philip L. De Jager, James K. Nuñez, Martin Kampmann

**Affiliations:** 1 Institute for Neurodegenerative Diseases, University of California, San Francisco, San Francisco, CA, USA; 2 UC Berkeley-UCSF Graduate Program in Bioengineering, University of California, San Francisco, San Francisco, CA, USA; 3 Department of Molecular and Cell Biology, University of California, Berkeley, Berkeley, CA, USA.; 4 Department of Electrical Engineering and Computer Sciences, University of California, Berkeley, Berkeley, CA, USA.; 5 Center for Translational & Computational Neuroimmunology, Department of Neurology & Taub Institute for the study of Alzheimer’s Disease and the Aging Brain, Columbia University Irving Medical Center, New York, NY, USA.; 6 Engineering and Technology Department, City College of San Francisco, San Francisco, CA, USA; 7 Chan Zuckerberg Biohub, San Francisco, CA, USA; 8 Department for Biochemistry and Biophysics, University of California, San Francisco, San Francisco, CA, USA; 9 Lead contact

## Abstract

Microglia, the brain’s innate immune cells, can adopt a wide variety of activation states relevant to health and disease. Dysregulation of microglial activation occurs in numerous brain disorders, and driving or inhibiting specific states could be therapeutic. To discover regulators of microglial activation states, we conducted CRISPR interference screens in iPSC-derived microglia for inhibitors and activators of six microglial states. We identified transcriptional regulators for each of these states and characterized 31 regulators at the single-cell transcriptomic and cell-surface proteome level in two distinct iPSC-derived microglia models. Finally, we functionally characterized several regulators. *STAT2* knockdown inhibits interferon response and lysosomal function. *PRDM1* knockdown drives disease-associated and lipid-rich signatures and enhanced phagocytosis. *DNMT1* knockdown results in widespread loss of methylation, activating negative regulators of interferon signaling. These findings provide a framework to direct microglial activation to selectively enrich microglial activation states, define their functional outputs, and inform future therapies.

## Introduction

Microglia are highly plastic brain-resident macrophages that continuously monitor the parenchyma to support cognitive function and brain health^1,2^. In response to insult or injury, microglia rapidly trigger transcriptional activation programs, or states, that allow them to restore homeostasis. Advances in single-cell technologies have markedly expanded our understanding of the diversity of microglial activation states^3^. Along with the discovery of new activation states, these data enabled identification of specific states that are enriched or depleted in disease conditions^4-7^. In rare cases, these activation states have been linked directly to expression of disease risk-associated genes, highlighting the importance of these states for brain health^4,5,8,9^.

The disease-associated microglial (DAM) activation state has been shown to increase during aging and is further expanded in neurodegenerative diseases including Alzheimer’s disease, Parkinson’s disease, Frontotemporal dementia, and Amyotrophic Lateral Sclerosis^5,6,10-12^. This activation state has been most deeply studied in the context of Alzheimer’s disease where DAM cells cluster around beta-amyloid plaques and contribute to their compaction and clearance^13-17^. The Alzheimer’s disease risk gene Triggering Receptor Expressed on Myeloid cells 2 (*TREM2*) is a key regulator of this activation state. Limiting DAM activation is hypothesized to be the mechanism whereby TREM2 mutations affect disease risk^16,18-21^.

Lipid-rich microglia have also been associated with Alzheimer’s disease as well as multiple sclerosis, neuropathic pain, and stroke^8,22-26^. Microglia containing lipid droplets are thought to represent a highly phagocytic state, clearing myelin and dead cell debris. Whether the lipid droplets themselves represent a beneficial sequestration of excess lipids or induce dysfunction is still debated and may differ depending on the disease, though there is evidence that reducing lipid droplets may be beneficial^25,27,28^.

Interferon-responsive microglia are activated in response to viral infections such as HIV-associated neurocognitive disorders^29^ but are also present in sterile conditions, particularly during developmental windows synaptic refinement^30^. Interferon-responsive microglia have been associated with developmental conditions including autism spectrum disorder^29-31^, but are also present in aging, multiple sclerosis, and tauopathies, where these cells induce aberrant synaptic pruning and contribute to neuroinflammation^32-36^.

Antigen-presenting microglia interact closely with T cells, a disease mechanism increasingly recognized in Alzheimer’s disease and related dementias^37-40^. Chemokine-high microglia are less well understood but have been profiled in Alzheimer’s disease, FTD/ALS, and autism spectrum disorders^41-43^. Increased chemokine secretion may impact infiltration of peripheral immune cells into the brain^38,44^ and has also been shown to increase microglial surveillance^18,45-47^. Lastly, the activation of many of these states is concordant with decreases in homeostatic microglia markers, though it is not known whether this decrease is necessary to permit activation or a separate but often convergent pathway. Homeostatic microglia express high levels of purinergic receptors that are used to surveille the brain environment.

Despite the critical importance of microglial activation states to their physiological function, we do not understand the transcriptional regulation of these activation states. The ability to selectively drive or inhibit microglial states will enable their functional characterization, support our understanding of how specific states contribute to disease, and enable the reprogramming of these states for therapeutic purposes.

Gene regulatory network (GRN) prediction algorithms such as SCENIC^48^ have been applied to nominate regulators of specific states with some success^49-55^. However, GRN prediction algorithms are still being refined to optimize accuracy, and all predictions need to be functionally validated. Furthermore, these algorithms rely on predicted transcription factor binding motifs. Many of these motifs have never been functionally validated. Additionally, for ~40% of transcription factors, DNA consensus sequences have not been identified^56^, limiting the power for GRN prediction algorithms to nominate these regulators. To overcome these challenges, we directly tested transcription factor (TF) and transcriptional regulator perturbation to determine regulators of six microglial activation states: homeostatic, disease-associated (DAM), lipid-rich, antigen-presenting, interferon-responsive, and chemokine. We performed these screens in iPSC-derived microglia because this model recapitulates a heterogeneous population of microglial activation states at baseline allowing for discovery of both drivers and inhibitors of each state^57^. The results of our screens showed some overlap with predicted regulators^51,54,55^, and additionally uncovered many novel regulators of microglial activation states.

We selected 31 dynamic regulators from the six large-scale CRISPRi screens to investigate more deeply at the single-cell level by combining CRISPR-droplet RNA-sequencing (CROP-seq) with Cellular Indexing of Transcriptomes and Epitopes sequencing (CITE-seq). To increase the robustness of our findings, we performed these single-cell based studies using two microglia differentiation protocols^57,58^, and observed that each model exhibits distinct activation profiles at baseline. This observation highlights the importance of selecting appropriate models that recapitulate the disease state or activation trajectories of interest.

Lastly, we generated individual CRISPRi knockdown cell lines for Signal Transducer and Activator of Transcription 2 knockdown (*STAT2*), PR-domain zinc finger protein 1 (*PRDM1)*, and DNA methyltransferase I (*DNMT1)*. We highlight marked decreases in interferon-responsive microglia for *STAT2* and *DNMT1* KD cells, though this impact of *DNMT1* KD is paired with increases in homeostatic and lipid-rich signatures. We highlight a potential mechanism of interferon-response inhibition through USP18/ISG15 by enzymatic methylation sequencing. Lastly, we highlight *PRDM1* as a tunable negative regulator of the the disease-associated (DAM), antigen-presenting, and lipid-processing states. We quantified strong up-regulation of these gene signatures and related functions in *PRDM1* KD microglia.

Together, our data represent the first large-scale perturbation study to determine regulators of microglial activation states. We highlight relationships between activation trajectories, discuss differences across two microglial models, and provide multi-modal validation of the impact of these perturbations on microglial gene expression, protein expression, and function. We anticipate that this dataset will provide a valuable resource to isolate and enrich microglial activation states, explore their downstream effects, and guide future mechanistic and translational research.

## Results

### CRISPRi screens uncover regulators of microglial activation states

**Figure 1.**
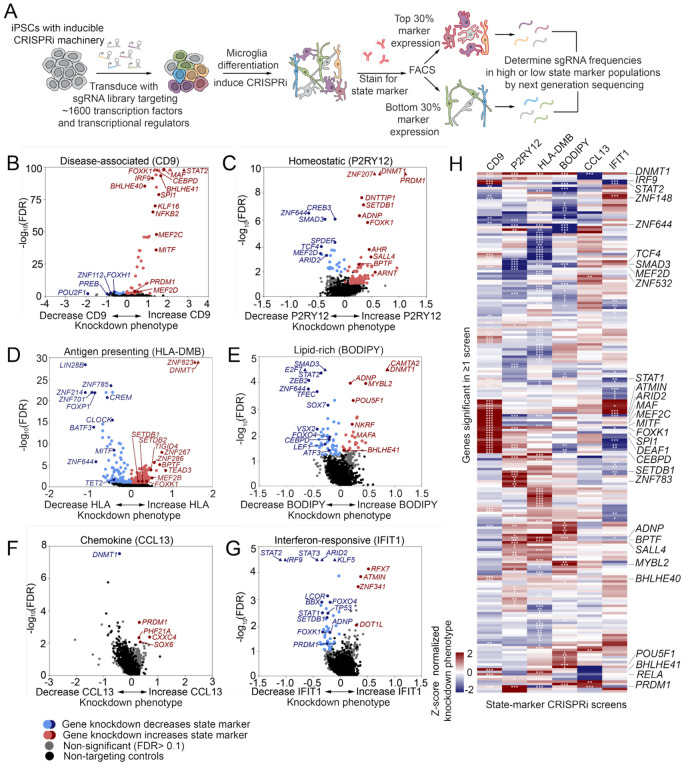
CRISPRi screens uncover transcriptional regulators of microglial activation states (A) Schematic of FACS-based CRISPRi screens. iPSCs expressing inducible CRISPRi machinery were transduced with a pooled library of sgRNAs targeting ~1600 transcription factors and transcriptional regulators. iPSCs were differentiated into microglia (iTF-MG protocol) and CRISPRi knockdown was induced with trimethoprim. Microglia were stained with markers for six activation states. The 30% of cells with the lowest and highest marker expression were separated by FACS. Genomic DNA was isolated from each population and the cassette encoding sgRNAs was amplified and sequenced using next-generation sequencing. Comparison of the frequencies of cells expressing each sgRNA to those expressing non-targeting control sgRNAs was used to determine hits. (B-G) Volcano plots of screening results for each state. Knockdown phenotype is calculated as a log_2_ ratio of counts in the high and low marker expression populations normalized to the standard deviation of non-targeting control cells. CRISPRi targets that significantly (FDR < 0.01) increase or decrease state marker expression are colored in red or blue, respectively. Gene knockdowns that do not significantly alter state marker expression (FDR ≥ 0.01) are shown in grey. Quasi-genes generated from random sets of non-targeting control sgRNAs to model a null distribution are shown in black. (H) Heatmap representing screen results for all genes (rows) that were significant in one or more of the screens shown in H. Each column represents an individual screen. Color scale represents the knockdown phenotype and significance represents FDR. Labelled genes were selected for secondary screens with deep single-cell phenotyping. *FDR < 0.05, **FDR < 0.01, ***FDR < 0.001 FDR.

To determine transcriptional regulators of microglial activation states, we performed six fluorescence-associated cell sorting (FACS)-based CRISPR interference (CRISPRi) screens using a single guide RNA (sgRNA) library targeting ~1600 transcription factors and transcriptional regulators (Figure 1A). In this library, each gene was targeted by five independent sgRNAs to control for off-target effects and 250 non-targeting control sgRNAs to control for all sources of noise. We selected markers for six activation states that have been reported^3,40,41,54,57,59-61^ to impact brain homeostasis and disease risk: Homeostatic (marked by P2RY12), disease-associated (DAM, marked by CD9), antigen-presenting (marked by HLA-DMB), lipid-rich (marked by BODIPY, neutral lipid dye), chemokine (marked by CCL13), and interferon-responsive (marked by IFIT1).

We previously developed a transcription factor (TF)-directed microglia differentiation protocol (iTF-MG) that reduces the bottlenecking of sgRNA libraries that occurs in more complex differentiation protocols^57^. iTF-MG were differentiated and stained for each of the state markers described above independently (P2RY12, CD9, HLA-DMB, BODIPY, CCL13, IFIT1) and sorted using FACS for high (top 30%) or low (bottom 30%) expression of each marker (Figure 1A). Enrichment of cells expressing each sgRNA in the high and low populations was quantified and compared against cells expressing non-targeting control sgRNAs.

The screen based on expression of CD9 (disease-associated microglia, DAM) uncovered a strong skew of positive regulators with 50 genes for which knockdown increased CD9 expression and 8 genes for which knockdown decreased CD9 expression (Figure 1B, Table S1). This suggests that many transcriptional regulators actively suppress the disease-associated (DAM) signature. Indeed, we found that many of our positive hits had been previously identified to be enriched in human postnatal microglia over fetal microglia (*PRDM1, SPI1, IRF1, KLF2, KLF6, KLF8, KLF16*)^62^. The DAM state is not highly expressed in healthy adult microglia, so this overlap supports our hypothesis that the DAM state may be actively suppressed. Comparing our functional data to previous predicted regulators of the DAM state^14,53,55^, we report that perturbation of the following predicted regulators does indeed alter expression of CD9: *BLHLE40, BHLHE41, SPI1, MAF, MEF2C, MITF, STAT1, CEBPD, KLF6*.

Screening based on P2RY12 expression (homeostatic state) identified 80 genes for which knockdown increased P2RY12 and 20 genes for which knockdown reduced P2RY12 (Figure 1C, Table S1). This bias towards discovery of genes for which knockdown increases P2RY12 is not surprising, given that the iTF-MG microglia model used for these screens has relatively low levels of P2RY12 at baseline, although an updated media composition we developed for these experiments does increase P2RY12 expression (Figure S1). These results highlight additional transcription factors that could be activated to further increase homeostatic markers including SMAD3, which functions downstream of TGFB1, a key homeostatic signal for microglia^63^.

Screening based on HLA-DMB expression (antigen-presenting state) produced the highest number of hits, identifying 197 genes for which knockdown increased HLA-DMB and 88 genes for which knockdown reduced HLA-DMB (Figure 1D, Table S1). Of interest, this screen revealed a potential connection between regulators of DNA methylation and expression of HLADMB. One DNA methyltransferase (*DNMT1*) and several readers of DNA methylation (*SETDB1, SETDB2, MECP2*) were all positive hits in the screen. Additionally the transcription factor *YY1*, that stabilizes DNA methylation^64^, was also a positive hit. Further corroborating this relationship, a DNA demethylase (*TET2)* was a negative hit. Together, this data suggests that hypo-methylation of DNA in microglia may increase antigen presentation. Studies of various cancer tissues have also highlighted this relationship using chemical inhibitors of DNMT1^65,66^.

Screening based on BODIPY staining (lipid-rich state) revealed 46 negative hits for which knockdown reduced lipid load and 23 positive hits for which knockdown increased lipid load (Figure 1E, Table S1). Interestingly, we found that 14 genes overlap as hits in both the CD9 and BODIPY screens with ~75% impacting these states in opposing directions (Figure 1E, Table S1). This may suggest a relationship between the two activation states, possibly reflecting increased lipid droplet clearance in disease-associated microglia. Expression of *CEBPD*, for example, is known to promote lipid accumulation in macrophages^67^, which our knockdown screen confirmed in microglia, and was also determined to be a regulator of the disease-associated state in our CD9 screen (Figure 1B).

The screen based on CCL13 expression (chemokine state) revealed only 4 hits (Figure 1F), suggesting a limited ability to alter this state by transcription factor perturbation, which is compatible with previous reports that chemokine expression is tightly regulated by post-translational signaling^68,69^. Interestingly, three out of the four genes for which knockdown increased CCL13 (CXXC4, PHF21A, SOX6) have been linked to neurodevelopmental disorders, though their impact is primarily hypothesized to occur in non-microglial cell types^70-72^.

Screening based on expression of IFIT1 (interferon-responsive state) was performed with a pre-stimulation of IFNβ to induce microglia to enter the interferon-responsive state. This screen revealed an enrichment of negative regulators with 44 hits for which knockdown decreased interferon-response and 4 hits for which knockdown increased interferon response (Figure 1G, Table S1). The iTF-MG microglia model is known to have high levels of interferon signaling at baseline^57,73^; however, our recent full genome-wide screen for this state produced a more even distribution of positive and negative regulators^73^. As expected, we found that the interferon-stimulated gene factor 3 (ISGF3) complex consisting of *STAT1, STAT2,* and *IRF9* are all strong negative hits in this screen as these TFs are key amplifiers of type I interferon signaling. We also confirmed our previous discovery that knockdown of the ASD-risk gene Activity-Dependent Neuroprotector Homeobox (*ADNP*) biases microglia away from the interferon-responsive state^74^. Furthermore, we uncovered Tumor Protein 53 (*TP53*) as a regulator of interferon-responsive microglia. The relationship between interferon signaling and TP53 has been explored in the context of cancer^75,76^ and models of Down syndrome and Alzheimer’s disease, where blocking interferon signaling has been shown to limit senescence^77^. Our data suggests a reciprocal relationship where *TP53* expression also regulates interferon responses.

Considering the hits across all six screens collectively, we find that 65% of the significant regulators impacted an individual activation state, while 34% impacted two or more states (Figure 1C). Intriguingly, two factors impacted 5 out of 6 states: DNA methyltransferase I (*DNMT1*) and PR domain zinc finger protein 1 (*PRDM1*) (Figure 1H).

### Single-cell RNA-sequencing highlights distinct state landscapes across iPSC-microglia models

**Figure 2.**
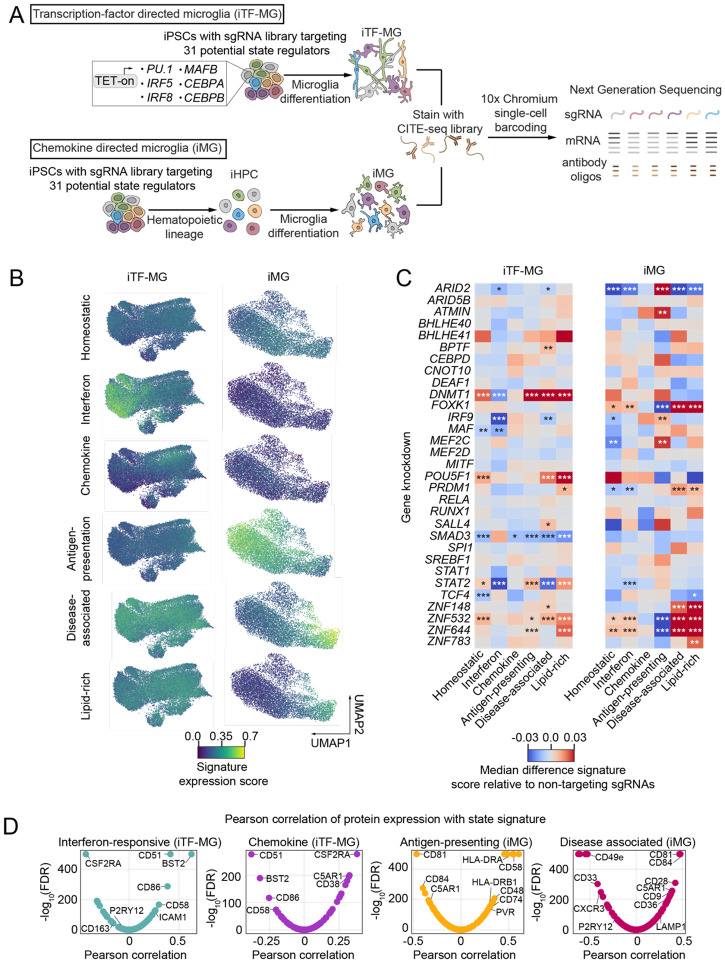
Single-cell RNA-sequencing highlights distinct state landscapes across iPSC-microglia models. (A) Schematic of the paired CROP-seq and CITE-seq experiments. iPSCs expressing inducible CRISPRi machinery were transduced with a pooled library of sgRNAs targeting 31 transcription factors and transcriptional regulators. 2 sgRNAs per gene, 5 non-targeting control sgRNAs. iPSCs were differentiated into microglia using the iTF-MG protocol from Dräger et al. 2022^57^ (top) or iMG protocol from McQuade et al. 2018^58^ (bottom). After differentiation, microglia were stained with a pooled oligo-tagged antibody library from Haage et al. 2025^79^ before single-cell barcoding with 10x Chromium and next generation sequencing of the sgRNA protospacer, mRNA, and antibody oligonucleotides. (B) UMAP representation of iTF-MG (left) and iMG (right) mRNA space. Cells are pseudocolored by state signature expression scores calculated by UCell. (C) Heatmaps of median difference in signature expression scores relative to non-targeting control cells calculated for each gene knockdown (rows) for each state (columns). *FDR < 0.05, **FDR < 0.01, ***FDR < 0.001 Mann Whitney U-test. (D) Pearson correlation of protein expression from CITE-seq analysis and mRNA expression from CROP-seq analysis. Correlation for interferon-responsive and chemokine states is highlighted in iTF-MG microglia. Correlation for antigen-presenting and disease-associated states is highlighted in iMG.

While the six marker-based screens are informative to nominate regulators of microglial activation states, an individual protein marker is often not sufficient to represent the full state. It is possible that some of the regulators in our screens increase only the state marker protein and not the full signature of proteins associated with a given state. To address this caveat, we conducted secondary screens using CRISPR-droplet RNA-sequencing (CROP-seq) to reveal the full transcriptomic changes downstream of 31 regulators selected from the primary screen hits (labelled in Figure 1C). For this targeted library, we included two sgRNAs for each gene target and five non-targeting sgRNAs. As there are clear differences between mRNA and protein expression, particularly in microglia^78^, and surface receptor expression is an important indicator of activation, we also profiled these cells by Cellular Indexing of Transcriptomes and Epitopes sequencing (CITE-seq) using an antibody library enhanced for the study of immune cells and microglia^79^ (Figure 2A).

Our large-scale transcription factor screens (Figure 1) revealed asymmetric discovery rates that appear to be dependent on the baseline activation state of the iTF-MG model (low homeostatic, low disease-associated, high interferon). For this reason, we performed our CROP-seq and CITE-seq analysis in two distinct iPSC-derived microglia protocols: transcription-factor driven differentiation (iTF-MG)^57^ and chemokine-driven differentiation (iMG)^58^. While both models express high levels of microglia identity genes including *IBA1 and CSF1R* (Figure S2), each differentiation protocol resulted in a unique composition of baseline activation states (Figure 2B).

Traditional clustering algorithms assume that individual cells must occupy one distinct state, however this is not aligned with our current understanding of microglial activation. Modeling microglial states as independent factors that can combine modularly is a better representation of the underlying biology^80,81^. Thus, we utilized an activation state signature curated from the literature to define gene expression profiles for each state (Table S2). Mean expression scores for these signature gene sets were calculated for every cell using UCell. Based on these signature scores, iTF-MG show a clear separation between cells in an interferon-responsive state and chemokine state (Figure 2B), corroborating our previous single-cell studies using this model^57,74^. Additionally, we find that disease-associated (DAM) and lipid-high signatures are high across most iTF-MG cells while antigen-presenting genes are uniformly low (Figure 2B). In the chemokine-driven iMG model, we find an activation axis of disease-associated (DAM) and lipid-high to homeostatic to antigen-presenting states. In the absence of stimulation, the iMG model shows exceptionally low levels of interferon-responsive and chemokine states (Figure 2B).

Since our initial screens were based on a single protein readout, we expected that a subset of the hits from these screens would not affect the full transcriptomic state signature. Indeed, knockdown of several hits did not significantly alter any of the states we define in this study (Figure 2C). These genes may be responsible for partial transcriptomic signatures (Table S4) or perhaps act on the state marker proteins through mechanisms unrelated to microglial activation.

Interestingly, many transcription factor perturbations produced distinct activation profiles in the two microglia models. For example, while *STAT2* knockdown decreased the interferon-response state in both iTF-MG and iMG, *STAT2* knockdown also had broader effects on multiple states in iTF-MG that were not identified in iMG (Figure 2C). Similarly, while *SMAD3* knockdown decreased homeostatic state genes in iTF-MG, this trend was not significant in iMG (Figure 2C). Since our RNA-sequencing data highlighted better separation for chemokine and interferon-responsive states in the iTF-MG model and better separation for DAM and lipid-rich states in the iMG model, follow up studies into specific regulators were primarily performed using the method that best recapitulates each state.

Interestingly, in both microglia models, we found high concordance of the disease-associated (DAM) and lipid-rich mRNA signatures (Figure 2C). For iTF-MG, three of the six gene knockdowns that induced DAM also increased the lipid-rich signature. For iMG, five out of the six gene knockdowns that induced DAM also increased the lipid-rich signature. In our original transcription factor-wide regulator screens, we found a marked opposition between the results of the anti-CD9 (DAM) and BODIPY (lipid) screens (Figure 1B, E). This suggests that the lipid-rich gene signature may not always be correlated with high levels of lipid droplets. This data highlights the importance of confirming functional outcomes that are expected to be correlated with gene signatures as the lipid-rich microglia state is often assumed to represent cells with high lipid droplet load.

### Microglial activation states are correlated with distinct surface protein signatures

The microglia “sensome” is a collection of surface receptors microglia use to detect and react to environmental changes. Cell-surface protein expression is dynamically regulated as microglia activate and alter the functional properties of the cell. Merging our CROP-seq and CITE-seq analyses, we were able to correlate protein expression of 166 surface proteins with each of the microglia activation states (Figure 2D, Table S3). Intriguingly, these results corroborated the mRNA analysis, where iTF-MG showed distinct separation of interferon-responsive and chemokine states. At the protein level, proteins positively correlated with the interferon-response state (CD51, BST2, CD86, CD58, ICAM1, etc.) were negatively correlated with the chemokine state (Figure 2D). We found a similar relationship between the antigen-presenting and disease-associated states in iMG, with CD81, CD84, C5AR1, CD9, CD28 being positively correlated with the DAM state and negatively correlated with the antigen-presenting state (Figure 2D). The antigen-presenting state is also positively correlated with known antigen-presenting proteins including HLA-DRB1, HLA-DRA, CD58, CD48, CD74 etc. (Figure 2D, Table S3). These relationships were also found in primary human microglia isolated from surgical and post-mortem specimen by Haage et al. 2025^79^ which reports the design and deployment of a microglia-enhanced CITE-seq reagent. These results suggest that the relationships between surface proteins and activation states in our model systems reflect *ex vivo* relationships, particularly for the interferon-responsive, antigen-presenting, and DAM states. These data may also be used to assess novel relationships between surface protein expression and activation state.

### Loss of STAT2 inhibits the interferon-response state

**Figure 3.**
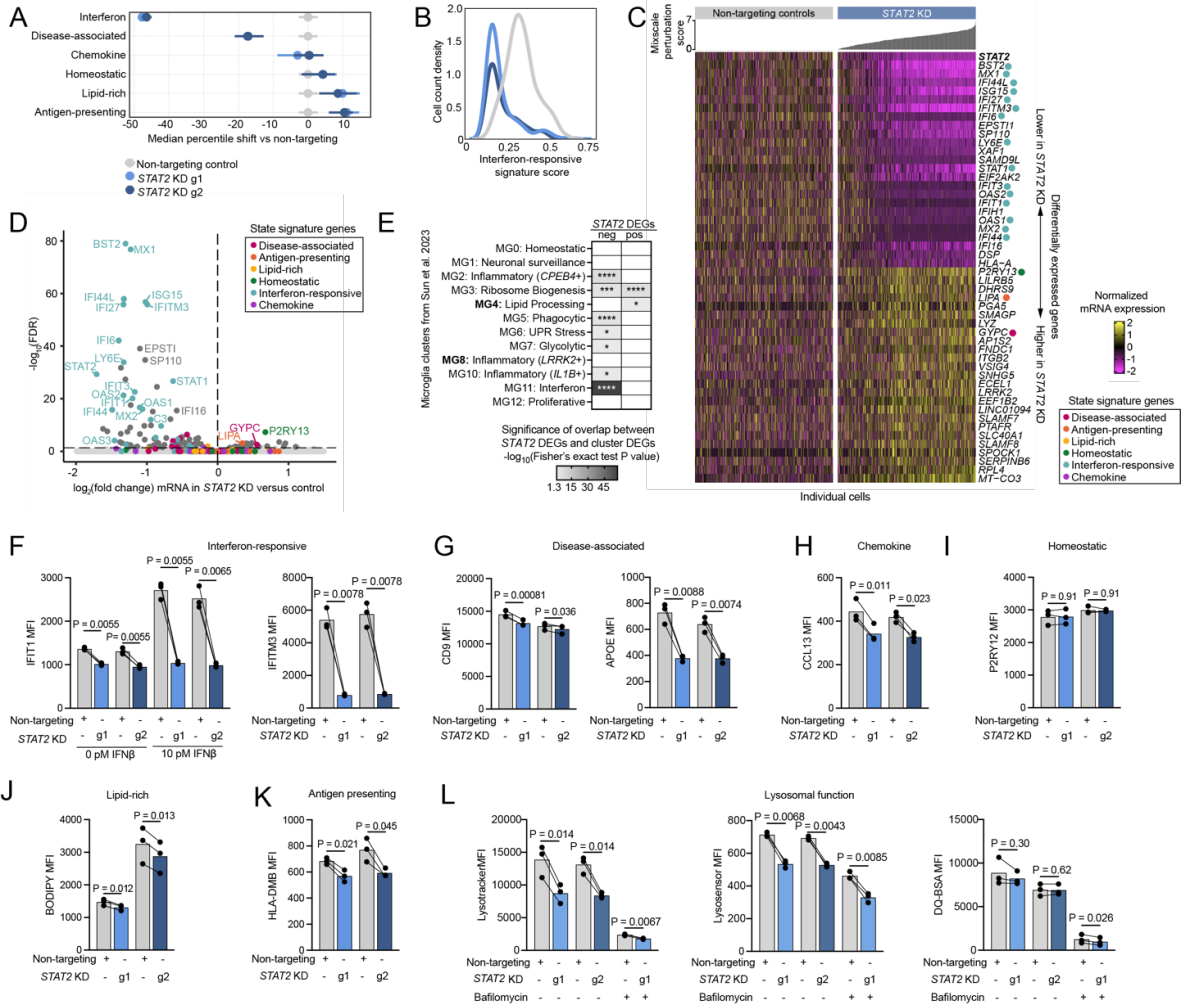
Loss of STAT2 inhibits the interferon-response state. (A) Median percentile shift of state signature scores in *STAT2* KD (blue) versus non-targeting controls (grey). Separate sgRNAs are represented as g1 and g2. Error bars represent 95% confidence interval. (B) Density plot of interferon-responsive signature score for *STAT2* KD (blue) versus non-targeting controls (grey). (C) Heatmap of top differentially expressed genes (DEGs) ranked by FDR (25 positive DEGs, 25 negative DEGs). Columns represent individual cells ordered by Mixscale perturbation score (see methods for details). Scores are graphed above the corresponding column (non-targeting control perturbation scores are all zero by definition). Color scale represents the log-normalized mRNA expression. DEGs that appear in our state signature scores are denoted with a colored dot (pink: disease-associated, orange: antigen-presenting, yellow: lipid-rich, green: homeostatic, teal: interferon-responsive, purple: chemokine). (D) Volcano plot of differentially expressed genes in *STAT2* KD versus non-targeting control. FDR ≤ 0.05. DEGs that appear in our state signature scores colored as above. (E) Heatmap of gene signature overlap analysis of DEGs from *STAT2* KD with human microglia clusters from Sun et al. 2023^54^. Bolded states are increased in AD patients. Color denotes significance of overlap by Fisher’s exact test P value. Non-significant overlap is white. *P < 0.05, **P < 0.01, ***P < 0.001 ****P < 0.0001. (F-K) Median fluorescence intensity (MFI) of state marker proteins by flow cytometry. *STAT2* KD microglia (blue) were compared to in-well non-targeting controls (grey) distinguished by nuclear fluorescent proteins. Each point represents an independent well, n ≥ 10,000 cells analyzed per well. P values from paired Student’s T-test. (L) Mean fluorescence intensity (MFI) of lysotracker, lysosensor, or DQ-BSA fluorescence in *STAT2* KD microglia (blue) and in-well non-targeting controls (grey). DQ-BSA was added for two hours before analysis to allow for uptake. Bafilomycin A was added four hours before analysis to control wells.

Our marker-based primary screens found Signal Transducer and Activator of Transcription 2 knockdown (*STAT2* KD) regulates lipid-rich, disease-associated, antigen-presenting and interferon-responsive states in the iTF-MG model. However, the full transcriptomic data highlights interferon-responsive genes as the main category of differentially expressed genes (DEGs) following *STAT2* knockdown (KD) (Figure 3A-D, Table S4). Interestingly, antigen-presenting and lipid-rich signatures were increased at the mRNA level in *STAT2* KD iTF-MG (Figure 3A) whereas staining for cell surface HLA-DMB protein levels or for lipid droplets using BODIPY confirmed the result of the original screens based on same antibody/dye (Figure 3J, K). This finding highlights the importance of multimodal analysis as opposed to identifying microglial activation profiles solely based on mRNA. Similarly, *STAT2* KD significantly decreased the disease-associated microglia mRNA signature, but only a subset of marker proteins (Figure 3G, Figure S3A).

To assess how our perturbations align with transcriptional signatures from human brain-derived microglia, we performed an analysis of the overlap of our gene sets with clusters identified from AD patient and control tissue from Sun et al. 2023^54^ (Figure 3E, Table S5). For genes decreased in *STAT2* KD microglia, we found strong overlap with clusters MG11, MG2, and MG5 from Sun et al. Cluster MG11 represents an interferon-responsive microglia cluster. MG2 represents a *CPEB4*-high inflammatory state the authors predicted to be regulated by IRF8. MG5 represents high expression of phagocytosis-related genes. For genes increased in *STAT2* KD microglia, we found significant overlap with MG3 and MG4. MG3 represents microglia high in ribosome biogenesis. MG4 represents lipid processing high microglia and is increased in AD patients.

To corroborate our screening data, we generated *STAT2* CRISPRi knockdown cell lines using two independent sgRNAs (g1 and g2) (Figure S3A). Activation state markers were quantified at the protein level by flow cytometry (Figure 3G-K). This confirmed that *STAT2* knockdown limits activation of interferon-responsive marker IFIT1 both at baseline and after stimulation with IFNβ (Figure 3F). We also find reduced levels of a separate marker of the interferon-responsive state, IFITM3 (Figure 3F). Additionally, we found lower levels of APOE, CCL13, HLA-DMB, and BODIPY staining in *STAT2* knockdown microglia (Figure 3G, H, J, K). P2RY12 and CD9 expression were not perturbed in *STAT2* KD microglia (Figure 3G, I). Additional protein markers of these states showed consistent results (Figure S4).

### *STAT2* KD decreases lysosomal markers

Microglia high in interferon-response are responsible for pruning neuronal synapses in development and hypothesized to do the same in disease. In epithelial cells, the interferon response increases lysosomal acidification and protease activity^82^, which are critical for phagocytosis. We assessed lysosomal properties in *STAT2* KD microglia to determine if tonic low-level interferon-response contributes to lysosomal function in microglia. We found that *STAT2* KD microglia did have reduced levels of pH-dependent lysosomal indicators (lysotracker and lysosensor), but found no difference in fluorescence of dye-quenched bovine serum albumin (DQ-BSA), a BSA conjugate that increases fluorescence after cleavage of quenching dyes by lysosomal cathepsins (Figure 3L).

### Loss of PRDM1 promotes the disease-associated microglia signature

**Figure 4.**
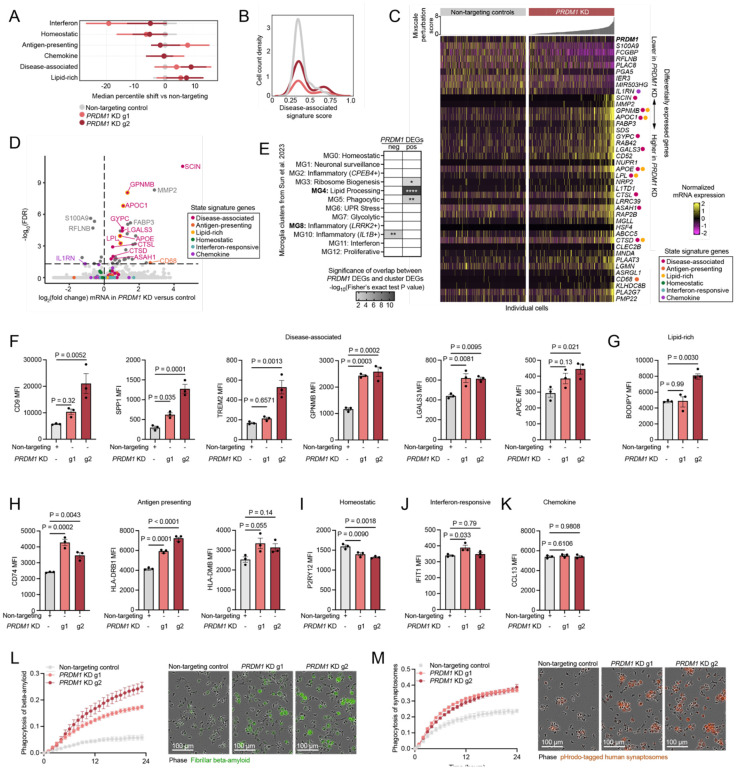
Loss of PRDM1 promotes the disease-associated microglia signature. (A) Median percentile shift of state signature scores in *PRDM1* KD (salmon, red) versus non-targeting controls (grey). Separate sgRNAs are represented as g1 and g2. Error bars represent 95% confidence interval. (B) Density plot of disease-associated signature score for *PRDM1* KD (salmon, red) versus non-targeting controls (grey). (C) Heatmap of top differentially expressed genes (DEGs) ranked by FDR (32 positive DEGs, 8 (all) negative DEGs). Columns represent individual cells ordered by Mixscale perturbation score (see methods for details). Scores are graphed above the corresponding column (non-targeting control perturbation scores are all zero by definition). Color scale represents the log-normalized mRNA expression. DEGs that appear in our state signature scores are denoted with a colored dot (pink: disease-associated, orange: antigen-presenting, yellow: lipid-rich, green: homeostatic, teal: interferon-responsive, purple: chemokine). (D) Volcano plot of differentially expressed genes in *PRDM1* KD versus non-targeting control. FDR ≤ 0.05. DEGs that appear in our state signature scores colored as above. (E) Heatmap of gene signature overlap analysis of DEGs from *PRDM1* KD with human microglia clusters from Sun et al. 2023^54^. Bolded states are increased in AD patients. Color denotes significance of overlap by Fisher’s exact test P value. Non-significant overlap is white. *P < 0.05, **P < 0.01, ***P < 0.001 ****P < 0.0001. (F-K) Median fluorescence intensity (MFI) of state marker proteins by flow cytometry. *PRDM1* KD microglia (salmon, red) were compared to non-targeting controls (grey). Each point represents an independent well, n ≥ 10,000 cells analyzed per well. P values from paired Student’s T-test. (L) Phagocytosis of fluorescently labelled fibrillar beta-amyloid in *PRDM1* KD (salmon, red) and versus non-targeting controls (grey). n=4 independent wells, 4 images per well. Points represent mean signal per well +/− standard error. Representative images of phase and fluorescent human synaptosomes (right). (M) Phagocytosis of pHrodo-tagged human synaptosomes in *PRDM1* KD (salmon, red) versus non-targeting controls (grey). n=4 independent wells, 4 images per well. Representative images of phase and fluorescent human synaptosomes (right).

PR-domain zinc finger protein 1 (PRDM1) is another regulator that impacts multiple activation states. The most highly impacted gene signatures in *PRDM1* KD versus control cells are the disease-associated (DAM) and lipid-processing activation states (Figure 4A-D). Although median percentile shift (Figure 1A) is more subtle, we find that *PRDM1* KD cells are bimodal for the disease-associated signature with a subset of cells showing very high DAM scores (Figure 1B). Weighted differential expression shows a strong enrichment of disease-associated microglia (DAM) genes up-regulated in *PRDM1* KD microglia (Figure 4C, D, Table S4).

One feature of targeting regulators with CRISPR interference rather than CRISPR knockout is that individual cells in the knockdown population may produce different levels of knockdown. For this reason, we performed weighted differential expression using Mixscale^83^ to identify lowly, moderately, and highly perturbed cells. This analysis revealed that *PRDM1* knockdown is not a simple on/off switch, and that expression of DEGs can be tuned based on the level of knockdown achieved with some genes only perturbed in the cells with the highest *PRDM1* KD score (Figure 4C, Table S4). We found that downregulation of negative DEGs already occurred at lower Mixscale scores (corresponding to weaker perturbation) than upregulated DEGs, which were observed only in cells with higher Mixscale scores. This may suggest that these downregulated DEGs are direct targets of PRDM1 as it is an inhibitory TF.

Comparing our *PRDM1* KD DEGs to human microglia, we unsurprisingly found that the up-regulated genes overlap with MG3, MG4, and MG5 from Sun et al. 2023^54^ which represent their ribosome biogenesis, phagocytic, and lipid-processing states (Figure 4E, Table S5). In their manuscript, Sun et al. highlight MG3 and MG4 as having significant overlap with the original DAM signature discovered in mouse by Keren-Shaul et al. 2017^4^. By comparing genes increased after *PRDM1* KD directly to the Keren-Shaul DAM signature, we also report significant overlap (P value = 1.1·10^−4^, Fisher’s exact test, Table S5). Our up-regulated genes additionally showed significant overlap with human AD-patient gene signatures as defined by Gerrits et al. 2021^60^. Specifically, their clusters 7, 8, 9, and 10 that are increased in AD patients and three of which correlated with beta-amyloid deposition in post-mortem tissue, are all significantly overlapping with *PRDM1* KD positive DEGs (P value for Cluster 7: 3.9·10^−6^, Cluster 8: 1.1·10^−10^ Cluster 9: 1.5·10^−6^, Cluster 10: 1.4·10^−10^ Fisher’s exact test, Table S5). We report very few genes with lower expression in *PRDM1* KD microglia (Figure 4D, Table S4). However, these genes are significantly overlapping with MG10 from Sun et al. 2023, an early inflammatory state high for IL-1β (Figure 4E, Table S5). Together these observations show a strong overlap of *PRDM1* DEGs with gene signatures relevant to Alzheimer’s disease progression and pathology.

We generated two independent *PRDM1* KD lines and found that one sgRNA (g2) reduced *PRDM1* expression to a greater extent than the other (g1) (Figure S3B). Using these lines, we found a strong increase in proteins associated with the DAM, lipid-processing, and antigen-presenting states (Figure 4F-H). Often g2 increased expression of these markers to a greater extent than g1, further supporting the notion that the downstream consequences *PRDM1* KD may be tunable. Additionally, we found no change in expression of IFIT1 and CCL13 at the protein level, and a modest decrease in P2RY12 (Figure 4I-K). Additional protein markers of these states show consistent results (Figure S5).

### *PRDM1* KD promotes phagocytosis of synaptosomes and beta-amyloid

Given the overlap of *PRDM1* KD up-regulated DEGs with gene signatures that are associated with phagocytosis and correlated with beta-amyloid pathology in patients, we assessed the phagocytic capacity of *PRDM1* KD microglia after exposure to beta-amyloid or human brain-derived synaptosomes. We find that *PRDM1* KD microglia take up more beta-amyloid and synaptosomes than control microglia (Figure 4L, M). Since the fluorescent signal we quantified could be caused by increased uptake of fluorescent substrates or decreased clearance, we assessed lysosomal function using Dye-Quenched Bovine Serum Albumin (DQ-BSA) and quantified no difference in cleavage and unquenching of the dye (Figure S5C). We also assessed lysosomal number, lysosomal pH, and the function of lysosomal cathepsins (Figure S5D-F). We found no difference in lysosomal number and a modest decrease in lysosomal pH, but no difference in the function of Cathepsin D or L. Cathepsin B activity and levels of LAMP1 were increased only in the more stringent *PRDM1* KD with g2. Together, these results suggest that the increased phagocytosis in *PRDM1* KD microglia is likely due to increased uptake of material and not due to impaired lysosomal degradation.

### Loss of DNMT1 is generally permissive to activation excluding the interferon-responsive state

**Figure 5.**
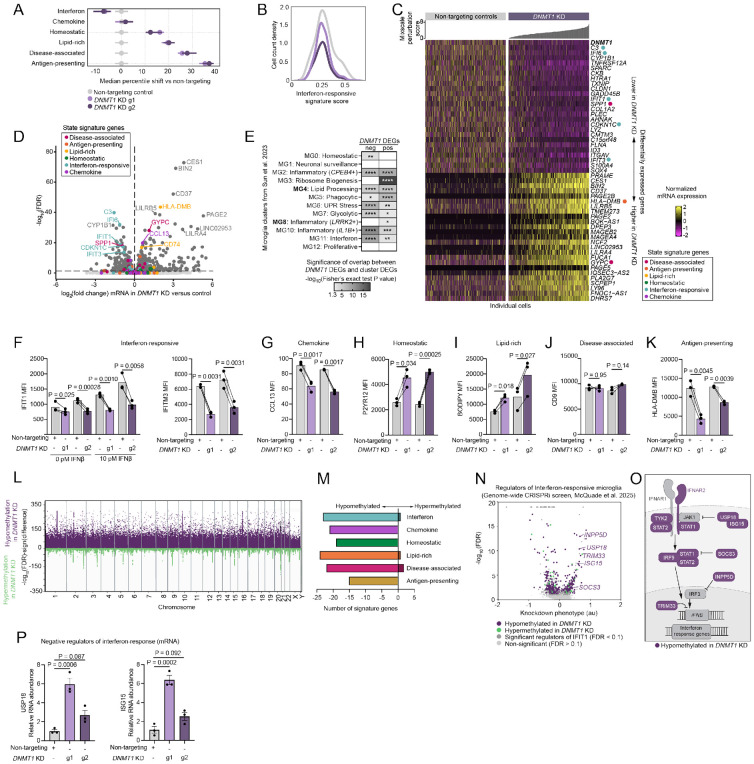
Loss of DNMT1 is generally permissive to activation excluding the interferon-responsive state. (A) Median percentile shift of state signature scores in *DNMT1* KD (violet) versus non-targeting controls (grey). Separate sgRNAs are represented as g1 and g2. Error bars represent 95% confidence interval. (B) Density plot of interferon-responsive signature score for *DNMT1* KD (violet) versus non-targeting controls (grey). (C) Heatmap of top differentially expressed genes (DEGs) ranked by FDR (25 positive DEGs, 25 negative DEGs). Columns represent individual cells ordered by Mixscale perturbation score (see methods for details). Scores are graphed above the corresponding column (non-targeting control perturbation scores are all zero by definition). Color scale represents the log-normalized mRNA expression. DEGs that appear in our state signature scores are denoted with a colored dot (pink: disease-associated, orange: antigen-presenting, yellow: lipid-rich, green: homeostatic, teal: interferon-responsive, purple: chemokine). (D) Volcano plot of differentially expressed genes in *DNMT1* KD versus non-targeting control. FDR ≤ 0.05. DEGs that appear in our state signature scores colored as above. (E) Heatmap of gene signature overlap analysis of DEGs from *DNMT1* KD with human microglia clusters from Sun et al. 2023^54^. Bolded states are increased in AD patients. Color denotes significance of overlap by Fisher’s exact test P value. Non-significant overlap is white. *P<0.05, **P < 0.01, ***P < 0.001 ****P < 0.0001. (F-K) Median fluorescence intensity (MFI) of state marker proteins by flow cytometry. *DNMT1* KD microglia (violet) were compared to in-well non-targeting controls (grey) distinguished by nuclear fluorescent proteins. Each point represents an independent well, n ≥ 10,000 cells analyzed per well. P values from paired Student’s T-test. (L) Manhattan plot showing CpGs of hypo- (purple) and hyper- (green) methylation from enzymatic methylation-sequencing. The x-axis is chromosomal location, and the y-axis is $-\log_{10}(\text{FDR})\times \text{sign}(\text{difference})$. (M) Classification of state signature genes for which the promoter region shows hypo- or hyper-methylation after *DNMT1* KD, The x-axis represents count of signature score genes in that category. (N) Volcano plot representing genome-wide CRISPRi screen for interferon-responsive microglia from McQuade et al. 2025^73^. Knockdown phenotype is calculated as a log_2_-ratio of counts in the high and low marker expression populations normalized to the standard deviation of the non-targeting control sgRNAs. Genes for which the promoter region shows hyper- or hypo- methylation are shown in green or purple respectively. Genes with no differential methylation pattern in *DNMT1* KD microglia are shown in grey. (O) Schematic of canonical Type I interferon signaling. Proteins colored in purple are hypo-methylated in *DNMT1* KD microglia and include three negative regulators: USP18, ISG15, SOCS3. (P) Reverse-transcription quantitative PCR analysis of *USP18* and *ISG15*. Points represent delta-delta Ct normalized to expression in non-targeting control cells. Error bars represent standard error.

DNA-methyltransferase I (DNMT1) is an epigenetic regulator that we found to strongly perturb microglial activation signatures. While *DNMT1* knockout is lethal during embryonic development^84,85^, using inducible CRISPRi to knockdown *DNMT1* during microglial differentiation avoids any toxicity (Figure S6). Knockdown of *DNMT1* enriched expression of all states except chemokine and interferon-responsive (Figure 5A). The interferon-responsive state signature was significantly decreased in *DNMT1* KD microglia (Figure 5A-D, Table S4).

The down-regulated DEGs in *DNMT1* KD had significant overlap with multiple human patient-derived microglial states as defined by Sun et al. 2023^54^. The strongest enrichment was for clusters MG10 and MG11 which represent inflammatory and interferon-responsive states (Figure 5E, Table S5). The up-regulated DEGs in *DNMT1* KD also overlapped with many human microglial states as may be expected due to broad hypo-methylation after loss of DNMT1 that leads to general increases in transcription. The strongest overlap was found with cluster MG3, a ribosome biogenesis state (Figure 5E). This state was also enriched for positive DEGs in *STAT2* KD microglia (Figure 3E), so it is possible that this phenotype is related to the inhibition of interferon-response as well.

In two independent *DNMT1* knockdown lines (Figure S3C), we confirmed that *DNMT1* KD inhibits the interferon response using multiple interferon-responsive proteins, IFIT1 and IFITM3 (Figure 5F). We additionally quantified a decrease in three markers of the chemokine state, which confirms our original screening results (Figure 1B, Figure 5G, Figure S6) but was not identified using mRNA-level analyses. We also confirmed increased levels of lipid-rich and homeostatic markers, consistent with both the original screens and mRNA analyses (Figure 5H, I). Markers of the DAM state were not consistently altered in *DNMT1* KD (Figure 5J, Figure S7). Lastly, we found a surprising decrease in antigen-presentation proteins (Figure 5K, Figure S7).

### Hypo-methylation in *DNMT1* KD enables expression of interferon-response inhibitors

Although DNMT1 is commonly thought of as a general DNA methyltransferase, it has also been shown to have specific activity guided by transcription factor binding^86,87^. Thus, we performed whole genome enzymatic methylation sequencing (EM-seq) in non-targeting control and *DNMT1* KD microglia to identify differentially methylated regions (DMRs) and determine if this is the mechanism whereby DNMT1 alters microglial activation. As expected, we found robust, widespread hypo-methylation in the *DNMT1* KD microglia (Figure 5L, Table S6). Hypo-methylation was also specifically present at promoters for the majority of genes in our state signatures (Figure 5M). This suggests that the decrease of interferon response in *DNMT1* KD is not due to conserved hyper-methylation at interferon response genes.

To nominate targets of DNMT1 that may be responsible for the inhibition of the interferon-responsive state, we mapped genes with hypo- or hyper-methylation onto our previous genome-wide CRISPRi screen for interferon-responsive microglia^73^. We found hypo-methylation at promoters of three negative regulators of interferon signaling (USP18, ISG15, SOCS3) (Figure 5N, O). Depending on the methylation unit, it is possible for transcription to increase or decrease^88^. We confirmed *DNMT1* KD microglia show increased mRNA levels of USP18 and ISG15 (Figure 5P). It is possible that *DNMT1* KD unleashes expression of interferon inhibitors leading to the robust decrease in interferon-responsive microglia that we reported.

## Discussion

In this study, we identified novel regulators for six microglial activation states through CRISPRi-based functional genomic screens. We perturbed ~1600 transcription factors and transcriptional regulators in iPSC-derived microglia and sorted microglia on expression of activation state markers for each state. For these large-scale, FACS-based screens, we relied on individual protein markers or dyes to classify microglial activation states. However, one individual protein does not capture the full state signature. Therefore, we selected 31 of these regulators to characterize at a deeper level by multimodal, single-cell analyses. Microglial activation states have been primarily defined at the transcriptomic level as advances in single-cell RNA-sequencing preceded other single-cell modalities. However, we know that there is often divergence between gene expression and protein expression. For this reason, we paired single-cell RNA-sequencing with a CITE-seq library of 166 surface proteins. This allowed us to correlate protein expression with the mRNA-based activation state signatures and uncover new surface markers for each state. Many of our state-associated proteins have been reported to be functionally relevant for that state, though future research is needed to understand if the novel correlations we report also contribute to state-specific functions.

We previously developed the iTF-MG microglia differentiation protocol to support large-scale CRISPR screening^57^. This model relies on doxycycline-inducible expression of PU.1, MAFB, CEBPA, CEBPB, IRF5, and IRF8 to directly drive microglial gene expression in twelve days. Importantly, using a directed differentiation approach avoids the bottlenecking events that occur in more complex chemokine-driven protocols such as iMG^58^. When differentiating iMG containing the full 9246 element sgRNA library targeting transcription factors and transcriptional regulators, we quantified dropout of >50% of sgRNAs throughout the differentiation. Thus, we performed the six large scale screens using the iTF-MG model. However, gene expression in the iMG model is known to align more closely with human patient-derived microglia samples. iMG also express higher levels of microglia identity genes (Figure S1) and homeostatic markers (Figure 2B) than iTF-MG. Therefore, for our targeted single-cell analyses, we used both iPSC-microglia models in parallel. With this smaller library, the iMG protocol still skewed representation of the sgRNAs from bottlenecking (Table S7), with ~ 50% of the sgRNAs assigned to less than 100 cells, although we were still able to perform meaningful statistical analyses for the majority of targeted genes by merging sgRNAs at the gene level. This study revealed distinct distributions of activation states present in each model. iTF-MG showed clear separation of the chemokine and interferon-responsive states while iMG showed clusters of disease-associated, lipid-rich, and antigen-presenting states. These results emphasize the importance of selecting the appropriate model system that reflects the biological states relevant to the research.

One outstanding question concerning microglial activation is whether these states represent mutually exclusive programs or can be modularly combined signatures. Our data supports the idea that individual cells can express multiple state signatures at once. Indeed, we identified many regulators that drive both disease-associated and lipid-rich states. However, we also reported anti-correlation between the interferon-responsive and chemokine states at the mRNA level, suggesting that some states may competitively interact. This type of interaction has been previously reported in our study of inflammatory states in astrocytes^89^. In future work, it will be important to investigate the functional relationships between microglial activation trajectories to understand the underlying molecular circuits that drive or limit activation plasticity. The regulators reported here build a foundational framework that would assist these deeper studies.

Following our multimodal sequencing analyses, we chose three regulators to study in independent knockdown lines: STAT2, PRDM1, and DNMT1. STAT2 is a well-studied transcription factor known to be required for type I interferon responses. Thus, we were not surprised to find that *STAT2* KD decreases interferon-response in both iTF-MG and iMG. However, STAT2 has not been widely investigated in microglia specifically, and we report for the first time that inhibition of *STAT2* causes lysosomal defects in iTF-MG. This is an important insight, since blocking microglial interferon responses is currently being investigated as a therapeutic mechanism for Alzheimer’s disease^35^. We also reported a strong decrease in APOE expression in *STAT2* KD microglia, an important finding given that APOE is one of the strongest risk factors for Alzheimer’s disease^90^.

PRDM1 is a transcriptional repressor that functions by recruitment of histone deacetylases (HDACs) to target genes. PRDM1 has been most widely studied in B cells where it is required for B cell maturation into plasma cells^91,92^, although PRDM1 is widely expressed across cell types. In microglia, PRDM1 expression is temporally restricted. PRDM1 is absent in microglia during development and activated as part of a postnatal microglia signature^62,93^. Interestingly, three recent studies have identified FDA-approved HDAC inhibitors as mimicking neuroprotective, DAM-like signatures^9,94,95^. As *PRDM1* relies on HDAC recruitment to repress targets, it is possible that *PRDM1* loss of function explains these findings.

In a study of APOE in iMG, *PRDM1* expression was shown to increase in response to neuronal conditioned media in APOE3/3 but not APOE4/4 microglia. These APOE3/3 microglia were also more responsive to stimuli and increased expression of DAM makrers such as GPNMB^96^. PRDM1 was also highlighted as part of a neuroimmune activation module along with the DAM gene *TREM2* in a study of tauopathies^97^. Here, we identified PRDM1 as a functional inhibitor of the DAM state and showed that knockdown of *PRDM1* is sufficient to drive expression of the core DAM signature genes. This makes PRDM1 an interesting therapeutic candidate to drive the DAM state in mature microglia, which is hypothesized to be beneficial for amyloid clearance in the context of Alzheimer’s disease^21,98^.

DNMT1 is a DNA methyltransferase primarily responsible for maintaining DNA methylation patterns during replication. Interestingly, DNMT1 has been shown to be necessary for hematopoietic development. Global *DNMT1* knockout mice are not viable, and *DNMT1* knockout specifically in the hematopoietic system leads to deficient maturation of hematopoietic stem cells^99^. Of note, from the five independent sgRNAs targeting DNMT1 used in our TF-wide screens, only three were present at the end of differentiation. It is possible that the two sgRNAs that dropped out had stronger knockdown of *DNMT1*, reducing viability. Indeed, the two sgRNAs targeting *DNMT1* that were used in our follow up studies have a modest ~50% knockdown of *DNMT1* and no toxicity.

Dysregulation of DNA methylation is a hallmark of aging and is known to contribute to age-related phenotypes including cognitive decline and AD^100-102^. In patients with Alzheimer’s disease, microglia specifically, show significant DNA hyper-methylation^103^. However, the impact of this hyper-methylation has not been investigated. Our functional genomic screens have identified that perturbing regulators of DNA methylation has a strong impact on microglial activation states. We specifically followed up on DNMT1, but determined that other regulators of DNA methylation also modify microglial activation (ex. TET2, SETDB1, SETDB2, CXXC4, MBD1, MBD6, Table S1). Guided by these results, we hypothesize that skewed microglial activation could explain the aging phenotypes correlated with altered DNA methylation in these microglia. Our study found hypo-methylation reduced interferon-responsive microglia. Further research is needed to understand if hyper-methylation increases interferon-responsive microglia. Since interferon-responsive microglia are known to remove synapses^30,34^, enriching this activation state could explain the correlation between hyper-methylation and Alzheimer’s disease.

An important property of CRISPR interference is that knockdown populations capture a spectrum of perturbation rather than the binary on/off signal from CRISPR knockout. This is beneficial as it reduces lethality that may occur from the complete loss of important transcription factors. We additionally leveraged this feature by applying Mixscale^83^ to rank cells based on perturbation strength. This also allows us to infer perturbation for genes that are expressed at too low a level to be quantified as a differential expression by single-cell RNA-sequencing. This analysis revealed interesting relationships between our regulators of interest and their downstream changes in gene expression. For *STAT2* KD, we find a binary switch where all DEGs were perturbed together and to the same extent above a Mixscale perturbation score of ~1.5 (Figure 3C). In contrast, *PRDM1* KD shows a more gradual change in gene expression suggesting that the impact of PRDM1 on the DAM state may be more tunable. We discovered a set of downregulated genes (S100A9, FCGBP, RFLNB) that are significantly changed even in cells with low Mixscale perturbation scores. We hypothesize that these genes may be direct targets inhibited by PRDM1. We also discovered a set of positive DEGs that turn on exclusively in cells with the highest perturbation scores (SCIN, MMP2, NUPR1). Many of our DAM genes also follow this trajectory with uniformly higher expression in the most highly perturbed cells. For *DNMT1* knockdown, the response of DEGs is more binary, likely due to widespread DNA hypo-methylation which increases as a function of cell divisions.

To contextualize our perturbations in iPSC-derived microglia with human brain-derived microglia, we performed overlap analysis of our differentially expressed genes from *STAT2*, *PRDM1*, and *DNMT1* knockdown with the human patient-derived microglia clusters identified in Sun et al. 2023^54^. In the original publication, the authors predict regulators of their microglial clusters based on transcription factor motif enrichment analysis. They predicted STAT2 to be a regulator of MG11, an interferon-responsive cluster. Our data confirms that STAT2 does indeed drive the genes defining this cluster. In contrast, the authors predicted PRDM1 to regulate clusters MG6 and MG7, but our data shows that PRDM1 DEGs only overlap with clusters MG3, MG4, MG5, and MG11. Instead, MG4, a lipid-processing state, shows the most significant overlap with *PRDM1* KD DEGs. Interestingly, their TF-enrichment analysis did not uncover any significant regulators for MG3, a ribosome biogenesis cluster. Our data reveals *DNMT1* to be a strong regulator of this signature. DNMT1 does not have a searchable DNA motif that can be utilized in these types of TF motif enrichment analyses, highlighting the benefit of our CRISPR screening approach and a broader focus on epigenetic regulators of gene expression beyond transcription factors.

Together, our data offer a unique lens to study microglial activation states. We importantly use multi-modal level analyses of state perturbations at the mRNA, protein, and functional level and highlight key cases in which there is misalignment between these modalities. This data strongly highlights the need to validate findings past the mRNA profiling stage and cautions against assuming downstream function based solely on mRNA expression.

### Limitations of the study

One limitation of this study is the use of iPSC-derived microglia models. In the brain, microglia are constantly signaling to their surrounding cells and receiving feedback from their environment. Microglial activation occurs and is resolved as a response to these complex combinatorial signals. By using iPSC-derived microglia in monoculture, we are not modeling this intricate environment. However, this platform provides the highest level of control and removes the confounding variables of environmental homeostatic or proinflammatory signals which may occur in *in vivo* models. Because the regulators we identified work through transcriptional regulation, we expect faithful translation *in vivo* is likely. However, further work will be needed to test if our regulators can override environmental signals.

Another limitation is that we were unable to perform the high-resolution single-cell analyses on the full ~1600 gene sgRNA library of transcription factors and transcriptional regulators due to the substantial cost of these experiments. For this reason, we opted to use a tiered approach with six unbiased screens for the ~1600 gene library, paired CROP-seq and CITE-seq for 31 hits nominated from the larger library, and detailed analysis of three genes that were particularly interesting by single-cell sequencing. While this approach ensured that our deeper characterizations focused on particularly robust regulators of microglial activation states, our data is necessarily limited in scope by the high cost of transcriptomic analysis.

## Methods

### iPSC maintenance

Human induced pluripotent stem cells were cultured in either StemFlex (Gibco, A33493-01, before transcription factor based differentiation protocol^57^) or mTeSR1 (Stemcell Technologies, 85850, before chemokine based differentiation protocol^58,104^). Cells were passaged once per week via ReLeSR (Stemcell Technologies, 100-0483). Human iPSC studies at the University of California, San Francisco were approved by the Human Gamete, Embryo and Stem Cell Research Committee.

### Transcription factor-driven differentiation of iPSC-Microglia (iTF-iMG)

iTF-MG were differentiated as previously described^57^ with a modified media composition optimized for increased expression of P2RY12, TREM2, and CD33. Induced pluripotent stem cells containing six doxycycline-inducible transcription factors: CEPBA, CEBPB, IRF5, IRF8, MAFB, PU.1, were passaged as a single-cell suspension using accutase (Thermo Fisher Scientific, A11105-01) for 7 minutes at 37 °C. Cells were plated to dual-coated Matrigel (Corning, 356231) and PDL plates (Corning, 356470) and cultured for 48 hours in Essential 8 (Gibco, A1517001) stem cell maintenance media supplemented with 2 μg/mL doxycycline (Clontech, 631311) and 10 nM Y-27632 (Tocris 1254). Two days post passage, cells were moved to microglia differentiation media: BrainPhys (StemCell Technologies, 05790), 0.5x N2 Supplement (Gibco, 17502048), 0.5x B27 (Gibco, 17504044), 10 ng/mL NT-3 (Peprotech, 450-03), 10 ng/mL BDNF (Peprotech, 45002), 1 μg/mL mouse laminin (Thermo Fisher Scientific, 23017-015) supplemented with 2 ug/mL doxycycline, 100 ng/mL IL-34 (Peprotech 200-34), and 10 ng/mL GM-CSF (Peprotech 300-03). On day 4, day 8, and day 12, media was replaced with microglia differentiation media supplemented with 2 μg/mL doxycycline, 100 ng/mL IL-34, 10 ng/mL GM-CSF, 50 ng/mL M-CSF (Peprotech 300-25), and 50 ng/mL TGFB (Peprotech100-21C). For experiments with CRISPRi, cells were supplemented with 50 nM trimethoprim (TMP) (MP Biomedical, 195527) every other day to maintain strong knockdown.

### Chemokine-driven differentiation of iPSC-Microglia (iMG)

iMG were differentiated as previously described^58,104^. iPSCs were passaged in small colonies ~50-100 cells onto Matrigel coated plates using ReLeSR (Stemcell Technologies, 100-0483) at a density suitable to achieve ~40 colonies per well the next day. Wells with the correct size and density of iPSCs were differentiated to hematopoietic progenitor cells (HPCs) using StemDiff Hematopoietic kit (Stemcell Technologies, 05310). HPCs were collected on days 10, 12, and 14 and frozen in BamBanker (Bulldog Bio, BB05). To generate iMG, HPCs were thawed at 7-10K cells/cm^2^ into microglia differentiation media: DMEM/F12, 2X insulin-transferrin-selenite, 2X B27, 0.5X N2, 1X glutamax, 1X non-essential amino acids, 400 μM monothioglycerol, 5 μg/mL insulin. Immediately before use, microglial medium was supplemented with 100 ng/mL IL-34, 50 ng/mL TGFei, and 25 ng/mL M-CSF (Peprotech) taken from single-use frozen aliquots. Media with fresh cytokines was supplemented 1 mL/well of a 6-well plate every 48 hours for 28 days.

### Lentivirus generation for individual sgRNAs

Lentivirus was generated as described^105^. HEK-293T cells were plated to achieve 80-95% confluence after 24 hours. For 2 mL of media, 1 μg transfer plasmid and 1 μg third generation packaging mix were added to 100 μL Optimem (Gibco, 31985088) and 12 μL TransIT-Lenti Transfection Reagent (Mirusbio, MIR 6604). Transfection mix was incubated for 10 min at room temperature before addition to cells. After 48 hours, conditioned media was collected and filtered through a 0.45 μm PVDF filter. Lentivirus Precipitation solution (Alstem, VC125) was used per manufacturer’s protocols to isolate lentivirus.

iPSCs were infected with lentivirus immediately after single-cell passaging with accutase and treated for 48 hours. After transduction, cells were again single-cell passaged and selected with 1 μg/mL puromycin for two passages or until >95% BFP positive populations were achieved.

### Pooled CRISPRi screening

For screens of transcription factors and transcriptional regulators, we used a previously developed^89^ sgRNA library targeting 1619 transcription factors and transcriptional regulators with five independent single guide RNAs (sgRNAs) per gene and 250 non-targeting sgRNAs. sgRNAs were chosen using the design of the Horlbeck et al. CRISPRi_v2 library^106^ and cloned into pLG15 vector^105^. To create lentivirus, HEK-293T cells were transduced as described above at an MOI of 0.3 to minimize multiple transduction of the same cell with multiple sgRNAs. To ensure equal distributions of gRNAs, transduction was performed at a coverage of 3000x such that every sgRNA would be represented in 3,000 iPSCs on average. For all cell culture, freezing, and differentiation, transduced iPSCs were maintained with at least 1000x average coverage of sgRNAs.

For each screen of microglial activation states, 20M iPSCs were differentiated using the iTF-MG protocol described above, with addition of TMP to induce CRISPRi activity starting at day 0. On day 12 of differentiation, iTF-MG were lifted with TrypLE for 10 min at 37 °C and pelleted at 300 xG for 5 min. To stain dead cells for removal, iTF-MG were resuspended in Zombie Red Fixable Viability kit (Biolegend, 423110, 1:200) for 10 min at 4 °C.

For intracellular markers (IFIT1, CCL13), iTF-MG were fixed with zinc fixation buffer (0.1M Tris-HCl, pH 6.5, 0.5% ZnCl_2_, 0.5% Zn Acetate, 0.05% CaCl_2_) overnight at 4 °C. Following fixation, cells were stained with anti-IFIT1 (1:100, 20329S, Cell Signaling) or anti-CCL13 (1:50, IC327G, R&D Systems) in TBS-based FACS buffer (TBS + 3% BSA + 0.5 uM EDTA) for 30 min at 4 °C. Cells were washed in TBS-based FACS buffer and strained through a 20 μm filter.

For extracellular markers, antibody was added to live cells in PSB-based FACS buffer (DPBS + 3% BSA + 0.5 μM EDTA) for 30 min at 4 °C. The following antibodies or dye was used: anti-CD9 (1:200, 312104, Biolegend), anti-P2RY12 (1:50, 392108, Biolegend), anti-HLA-DMB (1:200; 82922-1-RR Proteintech) BODIPY (1:1000, D3922, ThermoFisher). Following staining, cells were washed in PBS-based FACS buffer and strained through a 20 μm filter.

iTF-MG were sorted using a BD FACSAria Fusion flow cytometer. Cells were gated on live, single cells, and sorted into top 30% and bottom 30% of marker expression. After sorting, genomic DNA was isolated with NucleoSpin Blood L kit (Macherey-Nagel, 740954.20). sgRNA cassettes were isolated and amplified as previously described^105^. Screening libraries were sequenced on Illumina MiSeq.

### Single-cell CRISPRi screening (CROP-seq and CITE-seq)

Library Generation: The CROP-seq library was generated targeting 31 genes that had interesting phenotypes in the original six FACS-based screens. FACS-based screens were used to select the two highest-performing sgRNAs for each gene to include in the CROP-seq library. In total, 31 genes with 2 sgRNAs each were pooled alongside 5 non-targeting sgRNAs which did not influence phenotypes in any of the original FACS-based screens resulting in a final library size of 65 elements. The crop-seq library was prepared as previously described^57^ from arrayed oligonucleotides ordered from Integrated DNA Technologies. Top and bottom oligonucleotides for each sgRNA were annealed and then pooled. This pool was cloned into pMK1334 after restriction digest with BstXI and BlpI. The pooled plasmid library was cloned into Stellar Competent cells such that each plasmid is estimated to have been transformed into >500 bacteria. We performed sgRNA enrichment PCR and sequenced this plasmid pool using MiSeq to confirm normal distribution of all sgRNAs. Lentivirus was prepared and added to iPSCs as described above.

Cell preparation: iMG and iTF-MG containing the CROP-seq library were differentiated at >1000x coverage of the library. HPC differentiations were carried out at ~5000x to reduce the impact of bottlenecking at the HPC collection stage. iTF-MG and iMG differentiations were timed to reach day 12 and day 28 respectively on the same day. iTF-MG were lifted with TrypLE for 10 min at 37 °C. iMG are grown non-adherently and lifted in the media. Cells were treated with TruStain FcX Fc block (1:200, Biolegend, 422301) for 10 min prior to addition of CITE-seq antibody pools as described in Haage et al. 2025^79^. The Total-Seq A Universal (Biolegend, 399907) and custom^79^ antibody pools were centrifuged for 30 sec at 10,000 xG before reconstitution in 27.5 μL Cell Staining Buffer (Biolegend, 420201) total for both antibody panels and incubated at room temperature for 5 min. Before adding to iMG, the antibody pools were centrifuged for 14,000 xG for 10 min at 4 °C. 500,000 iMG and iTF-MG were incubated with the antibody panel for 30 min at 4 °C. After staining, cells were washed with 1 mL Cell Stating Buffer four times with centrifugation of 300 xG for 10 min. Cells were filtered with 40 μm filter, counted, and diluted to 1.5M cells per mL. Cells were loaded into the 10x Chromium Controller (10X Genomics v4) at 40,000 cells per lane for one lane per sample according to the manufacturer’s instructions. Single-cell sequencing libraries for CROP-seq were prepared as previously described^57^. CITE-seq libraries were prepared using ADT primers per manufacturer’s instructions. Libraries were sequenced on NovaSeq X 10B.

For iTF-MG, CROP-seq was performed with two biological replicates that were differentiated, stained, and sequenced separately and merged as described in the computational methods.

### Generating sgRNA knockdown lines

sgRNAs with strong phenotypes in the original screens were selected for follow up studies. sgRNAs were cloned into pMK1334 as previously described^74^. Individual gRNAs were added to iPSCs and selected as described above in lentivirus production. For samples with in-well non-targeting control, the plasmid backbone blue fluorescent protein (BFP) was swapped for mApple as previously described^74^. sgRNA sequences are listed in Table S8.

### Reverse-transcriptase quantitative polymerase chain reaction

To confirm RNA knockdown for independent sgRNA knockdown lines, RNA was extracted from mature microglia using the Quick-RNA Microprep Kit (Zymo, R1050). cDNA was generated using SensiFAST cDNA Synthesis Kit (Meridian Bioscience, BIO-65053). For quantitative PCR, SensiFAST SYBR Lo-ROX 2X Master Mix (Bioline; BIO-94005) was used with custom qPCR primers from Integrated DNA Technologies. Amplification was quantified using the Applied Biosystems QuantStudio 6 Pro Real-Time PCR System using QuantStudio Real Time PCR software (v.1.3). Fold change in RNA expression was calculated using ΔΔCt. Data normalized to *GAPDH* to control for RNA concentration.

### Flow cytometry

iTF-MG were lifted with TrypLE Gibco, 12604021) for 10 min at 37°C. iMG are grown non-adherent and do not require dissociation. For both cell types, iPSC-Microglia are pelleted at 300 xG for 5 min. iPSC-Microglia are resuspended in Zombie Live/Dead stain (1:200, Biolegend, multiple cat#) for 10 min at room temperature and washed in DPBS.

For extracellular markers, iPSC-Microglia are stained for 30 min at 4 °C in FACS buffer (DPBS + 3% BSA + 0.5 μM EDTA). Extracellular antibodies: anti-IFIT1 (1:100, 20329S, Cell Signaling), anti-CCL13 (1:50, IC327G, R&D Systems), anti-IFITM3 (1:200, CL488-11714, Proteintech), anti-APOE (1:200, MA5-15852, Invitrogen), anti-CCL2 (1:50, 125410, Biolegend), anti-CCL3 (1:200, 934603, Biolegend), anti-CD74 (1:50, 326808, Biolegend), anti-SPP1 (1:50, 50-9096-42, eBioscience), anti-TREM2 (1:100, MAB18281, R&D Systems), anti-CXCL10 (1:50, 519504, Biolegend), anti-LAMP1 (1:200, 328610, Biolegend), anti-APOC1 (1:200, PA5-145261, Invitrogen), anti-LIPA (1:200, 12956-1-AP, Proteintech).

For intracellular markers, iPSC-Microglia are first fixed with with eBioscience Intracellular Fixation and Permeabilization Buffer Set (Invitrogen, 888-8824-00) for 20 min at room temperature and washed in permeabilization buffer. antibodies are diluted in permeabilization buffer and added to cells for 30 min at 4 °C. Intracellular antibodies: anti-CD9 (1:200, 312104, Biolegend), anti-P2RY12 (1:50, 392108, Biolegend), anti-HLA-DMB (1:200; 82922-1-RR Proteintech), anti-LGALS3 (1:200, 125410, Biolegend), anti-GPNMB (1:200, 740041MP488, Invitrogen), anti-HLA-DRB1 (1:200, 327010, Biolegend).

For secreted markers, cells were pre-treated with GolgiPlug (1:1000, BD Biosciences, 555029) 6 hours prior to lifting.

Samples were analyzed on a BD FACSCelesta or BD LSR Fortessa X14 using BD FACSDiva software. Median fluorescence intensity was calculated using FlowJo analysis software after gating for live, single cells. Data was collected from 3 independent differentiations with 3 replicate wells per experiment (n ≥ 10,000 cells).

### Timelapse imaging phagocytosis assay

iMG were collected at day 28 and plated at 50% confluence on Matrigel-coated 96-well plates and allowed to recover overnight. After addition of pHrodo tagged human synaptosomes (10 ng/mL) or Alexafluor488-tagged beta-amyloid (2 μg/mL, Anaspec), images were collected every hour on IncuCyte S3 Live-Cell Analysis System (Sartorius) with 4 images per well in 4 independent wells per condition. IncuCyte 2023A software was utilized to mask fluorescent substrate signal normalized to cell body area.

### Flow cytometry analysis of lysosomal function

To analyze lysosomal function, control cells were pre-treated with Bafilomycin A (200 nM, Sigma, SML1661) for 4 hours. For uptake analysis, Dextran-Alexafluor488 10,000 MW (25 μg/mL, Invitrogen, D22910) or DQ Green BSA (10 μg/mL, Invitrogen, D12050) were added for 2 hours. For lysosome tracking, Lysotracker Deep Red (20 nM, Invitrogen, L12492) or Lysosensor Green (500 nM, Invitrogen, L7535) were added for 30 minutes. Samples were analyzed on BD FACSCelesta using BD FACSDiva software. Mean fluorescence intensity was calculated using FlowJo analysis software after gating for live, single cells. Data was collected from 3 independent differentiations with 3 replicate wells per experiment (n ≥ 10,000 cells).

### Cathepsin Activity assay

Activity of cathepsin B (Abcam, ab270772) and cathepsin L (Abcam, ab270774) was measured by fluorometric Magic Red cleavage assay per manufacturer’s instructions for three wells per cell line. Activity of cathepsin D was measured by fluorometric substrate cleavage kit (Abcam, ab65302) per manufacturer’s instructions for three wells per cell line. Fluorescence was read on Agilent BioTek Synergy H1 plate reader.

### TO-PRO viability assay

To assess viability, cells were treated with TO-PRO-3 (Thermo Fisher, R37113) and Hoechst (Thermo Fisher, R37165) for 15 min before imaging on IN Cell Analyzer 6000 (Cytiva). Images were analyzed using CellProfiler by masking all nuclei with the Hoechst, and performing a binary measurement of TO-PRO-3 negative (live) cells and TO-PRO-3 positive (dead) cells.

### Enzymatic-methylation sequencing

Whole genome enzymatic methyl sequencing (WGEMS) libraries were generated for DNMT1 knockdown or non-targeting control cells. Genomic DNA was isolated using genomic DNA was isolated with NucleoSpin Blood L kit (Macherey-Nagel, 740954.20). 200 ng of gDNA was diluted to 24 μL with TE buffer. Control DNA Unmethylated Lambda (NEB, E7122AVIAL) and Control DNA CpG methylated pUC19 (NEB, E7123AVIAL) were diluted 1:50 with TE buffer and 1 μL of each was added to the genomic DNA. All samples were sheared to 240-280 bp using NEBNext UltraShear^®^ (NEB, M7634L) following per manufacturer recommendations. End prep and adaptor ligation was performed using 44 μL of fragmented DNA following the NEBNext UltraShear^®^ manual (version 2.0, section 5). NEBNext^®^ Enzymatic Methyl-seq Kit (NEB, E7120S/L) was utilized using formamide denaturation for enzymatic methyl conversion. 5 ng of deaminated DNA was PCR-amplified for 6 cycles, followed by PCR-clean up and eluted in TE buffer according to protocol. Amplified libraries were quantified on an Agilent 2100 TapeStation system (Agilent). Samples were pooled at equimolar concentration and sequenced paired-end 150 bp reads on an Illumina NovaSeq X 10B. We obtained between 183.6M to 299M paired-end reads across all libraries.

### EM-sequencing analysis

Reads were trimmed using cutadapt (version 4.4)^107^, filtering empty resulting reads (-m 1) and specifying both forward (-a AGATCGGAAGAGCACACGTCTGAACTCCAGTCA) and reverse (-A AGATCGGAAGAGCGTCGTGTAGGGAAAGAGTGT) adapters. The resulting reads were aligned using Bismark (version 0.24.2)^108^ to the hg38 reference genome with methylated and unmethylated control DNA sequences provided by NEBNext (https://neb-em-seq-sra.s3.amazonaws.com/grch38_core%2Bbs_controls.fa). The Bismark tool deduplicate_bismark was used to deduplicate aligned reads. Next, bismark_methylation_extractor and bismark2bedGraph were then used to create a methylation report for CpG sites for the top and bottom strands. Additionally, coverage2cytosine was used to generate a cytosine report aggregating top and bottom strand reads. The top and bottom strand bedgraph files were combined (on the basis of CpG symmetry), and loci that had less than 3 reads after combining strands were filtered out. To ensure that samples within each condition were statistically independent, technical replicates for each sgRNA were combined by summing the total number of reads and number of methylated reads at each locus, resulting in one sample per sgRNA, for a total of two samples per condition.

Differential methylation analysis was performed using bsseq (version 1.40.0)^109^ and DSS (version 2.52.0)^110^ in R (version 4.3.3) by calling DMLtest with smoothing=TRUE. The analysis was performed between the non-targeting and the *DNMT1* KD cells each with two independent sgRNAs, for a total of 4 samples per differential analysis.

For aggregation, promoter regions were defined as +/− 500 bp from the TSS sites, and methyl sites were aggregated by mean difference of *DNMT1* KD vs non-targeting control, and selecting the minimum P value and minimum FDR. Significant regions were determined by minimum FDR < 0.05, and hypo- and hyper-methylated regions were determined by mean difference of less than 0 or greater than or equal to 0, respectively. Signature overlap was calculated by counting the number of genes for each signature that appear in the significant hypo or hyper-methylated regions. Overlap with the genome-wide interferon-responsive screen from McQuade et al . 2025^73^ was conducted by overlapping significant positive or negative DEGs with the significant hypo-methylated or hyper-methylated regions, with some genes labelled on the volcano plot.

### CRISPRi screen analysis

Analysis of CRISPR screens was as previously described^111^. Raw sequencing results were mapped with ‘sgcount’ (https://github.com/noamteyssier/sgcount). After, ‘crispr_screen’ (https://github.com/noamteyssier/crispr_screen) was used to perform differential enrichment analysis using a ‘drop first’ weighting strategy and Benjamini-Hochberg multiple hypothesis correction. A z score cutoff of 5 was implemented to remove spurious non-targeting control cells.

### Computational methods for single-cell analyses

Single-cell RNA Sequence Alignment: The raw single-cell sequencing reads of gene expression libraries were processed using kb-python (version: v0.29.1) from kallisto-bustools^112^. First, using the GRCh38 human reference transcriptome with Ensembl annotations (release 113), a kallisto index was generated with the default k-mer size (k=31). The 10x reads were then pseudo-aligned to this index using ‘kb count’ from kallisto-bustools with the ‘standard’ workflow and the cell barcode whitelist for Single Cell 3’ v3.1 chemistry.

Single-cell ADT Protein Assignment: The raw single-cell sequencing reads of antibody-derived tag (ADT) libraries were processed using the Cell Ranger software from 10x Genomics (version 9.0.0). The computational analysis was conducted on a high-performance computing (HPC) cluster configured with 48 GB of virtual memory and eight local cores. A custom feature reference file^79^ was used for detecting unique molecular identifiers (UMIs) associated with antibody-derived tags.

CRISPRi knockdown demultiplexing (sgRNA assignment): The raw sequencing reads of the PCR amplified sgRNA library were processed using kallisto-bustools. First, using `kb ref`, a kallisto index of the sgRNA library was generated with k-mer size of 9. The reads were then pseudo-aligned to this index using ‘kb count’ from kallisto-bustools with the ‘standard’ workflow and the cell barcode whitelist for Single Cell 3’ v3.1 chemistry. sgRNA assignment was then conducted using geomux (https://github.com/noamteyssier/geomux, version: v0.2.5), which conducts a hypergeometric test to calculate the significance of observing each sgRNA in each barcode. Cells were assigned to the majority sgRNA if they had a log-odds ratio above 1, Benjamini-Hochberg corrected P-value below 0.05, minimum UMI count to consider a barcode was 3, and minimum number of barcodes to consider a sgRNA was the default, 100.

Once cells were assigned to a sgRNA, the barcodes were used to intersect the gene expression and ADT count matrices to store both the RNA and protein expression counts for each sgRNA-assigned cell in MuData format from muon (version: v0.1.7)^113^. Cells with no barcodes or multiple barcodes were not included for further analysis. Both the iTF-MG and iMG samples were processed this way. Of the two biological replicates from iTF-MG samples, only one includes the CITE-seq library and was included for this additional analysis.

Quality Control: All QC was conducted using scanpy (version: v1.11.3)^114^. For both, the iTF-MG and iMG dataset with RNA and ADT counts, cells were filtered to include those with a minimum count of 200, and a mitochondrial count of less than or equal to 8%. Genes were filtered to include those with minimum cells of 10. For the iTF-MG experiment with only RNA counts, cells were filtered to include those with a minimum count of 1000. All other QC was conducted in the same way as the other datasets. For protein expression levels, four different normalization methods were initially tested and log_1_p-normalize was chosen as it had the highest median correlation to RNA expression as described in Haage et al. 2025^79^. Thus, for both RNA and protein expression levels, cells in each dataset were log_1_p-normalized to a target sum of 10,000. The top 5,000 highly variable genes were selected to conduct principal component analysis (PCA). Nearest neighbors were then calculated using the default 15 nearest neighbors, to then build UMAPs. Clustering was performed using different resolutions of the Leiden (version: v0.10.2)^115^ algorithm, and after setting the resolution to 1. Clusters with low *AIF1* expression (non-microglia cells) and low total counts were removed. 6 out of 18 clusters were removed from the iTF-MG replicate with only RNA data, whereas only 2 out of 13 clusters were removed from the iTF-MG replicate with both RNA and ADT data. Similarly, only 2 out of 9 clusters were removed from the iMG dataset. The two iTF-MG replicates were then concatenated and batch corrected using Harmony (version: v0.0.10)^116^, to get 3 final datasets used in analysis the iTF-MG and iMG MuData with both RNA and ADT counts, and the merged iTF-MG AnnData with the batch-corrected RNA counts. Two sgRNAs, FOXK1 sgRNA 2 (FOXK1_g2) and non-targeting control sgRNA 5 (non-targeting_g5) were also removed from analysis due to functional validation of FOXK1_g2 not producing knockdown (Figure S3H), and rigorous testing of non-targeting_g5 showing a perturbation effect through the Mixscale^83^ analysis described below. After QC, the resulting iTF-MG had 23,826 cells with RNA expression for 16,398 genes, and 9,040 cells with ADT expression for 180 proteins (166 proteins + 14 antibody controls). The iMG had 8,247 cells with RNA expression for 14,119 genes, and 8,247 cells with ADT expression for 180 proteins with 14 of those proteins included in the panel as controls.

### Mixscale Analysis:

#### Perturbation Score Calculation-

To quantify strength of knockdown, a custom-modified version of Mixscale (https://github.com/reetm09/mixscale) was used to get a Z-score normalized perturbation score for each cell. The modification replaces Mixscale’s Bonferroni correction method for multiple hypothesis correction with the Benjamini-Hochberg method in the initial calculation of differentially expressed genes to determine if a targeted knockdown had a perturbation effect. In Mixscale, this DEG effect vector is then used to calculate a per-cell perturbation score, taking advantage of the full transcriptome for assessing effect of knockdown, and providing a continuous score more representative of CRISPRi knockdown in comparison to binary classification of perturbed vs not-perturbed which is more representative of CRISPR knockout.

#### Weighted Differential Expression Analysis-

The per-cell perturbation score calculated above is used as a weight such that cells with higher perturbation scores (stronger knockdown effect) are given a higher weight into a negative binomial regression model for differential expression analysis. For perturbations that don’t have an initial DEG effect as classified by Mixscale, undergo standard differential expression analysis.

#### Visualization of Mixscale DEGs-

All visualizations of DEGs, including heatmaps and volcano plots, were conducted in R, utilizing the dpylr, ggplot2, ComplexHeatmap, and EnhancedVolcano packages. For heatmaps, individual cells (columns) were ordered by Mixscale score with a minimum score of 0, as depicted on histogram above heatmap. Each row depicts the log-normalized mRNA expression of the top 50 DEGs (top 25 downregulated and top 25 upregulated, FDR < 0.05). Non-targeting cells are unordered as they do not have Mixscale scores. For each perturbation, either non-targeting control or perturbed cells are downsampled to depict a balanced number of cells on the heatmap plot. Volcano plots are plotted with thresholds for significance at FDR < 0.05, with genes colored by signature.

Signatures Scores Analysis: Each signature was curated by literature (Table S2). Per-cell scores for each signature were calculated using a custom python implementation of the UCell^117^ algorithm, which is a robust method to calculate scores using Mann-Whitney U statistic test based on a ranked gene list. The signature score heatmap depicts median difference of signature scores between perturbations and non-targeting control cells with a Mann-Whitney U test for significance and FDR < 0.05 for identifying perturbations that significantly affect signatures. Median percentile shift point plots were made by converting the median difference into percentile shifts from non-targeting control cells for each signature. Error bars were calculated by 95% confidence intervals. Distributions of signature scores for each sgRNA were also plotted against non-targeting control cells after downsampling to the minimum number of non-targeting control or perturbation cells.

Protein-Signature Correlations: For each protein-signature pair, the Pearson correlation was computed by comparing the average protein level with signature scores for all cells. Protein names were paired with RNA names based on Ensembl gene IDs.

Overlap of Differentially Expressed Genes (DEG) with External Datasets: A Fischer’s exact test was used to test for significance when conducting an overlap of DEGs for each perturbation compared to external datasets pulled from literature. Significant DEGs were chosen with a FDR < 0.05.

## Supplementary Material

Supplement 1

2

Table S1. **Screen results for FACS-based screens.** Gene level results for all six screens. Each tab represents a separate CRISPRi screen: CD9, P2RY12, HLADMB, BODIPY, IFIT1, CCL13. Columns include fold change between high and low sorted cells (fc), log2fc, P value, FDR, and phenotype score which is the product of the log2fc and FDR.

Table S2. **State signature gene definitions.** Gene lists used for each state signature score. Each column represents a separate state.

Table S3. **Pearson’s correlation of CITE-seq proteins with state signature scores.** Each tab represents the data from a separate microglia differentiation protocol: iTF-MG and iMG. Within each tab the first column represents all 166 proteins and their correlation with each mRNA gene signature UCell scores. Values represent Pearson’s correlation.

Table S4. **Weighted differential expression for CROP-seq.** Each tab represents the data from a separate microglia differentiation protocol: iTF-MG and iMG. Columns represent perturbed gene targeted by CRISPRi, gene transcript name, log2fc, beta weight, P value, and the adjusted P value. Differentially expressed genes for all perturbations that were determined to be significant by Mixscale are included.

Table S5. **Overlap analysis with external data.** For STAT2, DNMT1, and PRDM1, positive and negative DEGs were compared to existing datasets. Columns represent the differentiation model, perturbed gene, direction of the DEG list analyzed (positive DEGs or negative DEGs), the cluster and paper of overlap, the number of genes overlapped between the DEGs and the cluster, the percentage of genes overlapped, the name of genes overlapped, and the fisher’s exact test P value.

Table S6. **Differential methylation at gene promoters from EM-seq.** Columns represent ensembl gene identification numbers, P value, FDR, and the mean difference where negative values are hypo-methylated in *DNMT1* KD cells versus non-targeting control guides.

Table S7 **Cell counts from CROP-seq analysis.** Number of individual cells assigned to each sgRNA. Columns represent the gene name and guide number, and the cell counts in iTF-MG or iMG models.

Table S8. **Protospacer sequences used in this study.** Protospacer sequences and sgRNA identities for for CROP-seq, CITE-seq, and downstream analyses. Columns: Gene name, guide number, protospacer name, protospacer sequence

## Highlights

- CRISPRi screening reveals novel regulators of six microglia activation states
- Multi-modal single-cell screens highlight differences between mRNA and protein level expression
- iPSC-microglia models show different baseline distributions of activation states
- Loss of DNMT1 leads to widespread DNA demethylation, promoting some states but limiting the interferon-response state
- Loss of PRDM1 drives microglial disease-associated state
