# Supplementary material for: Transcriptional regulation of disease-relevant microglial activation programs
Source: bioRxiv. 2025 Oct 27:2025.10.12.681832. Preprint. [Version 2] doi: 10.1101/2025.10.12.681832 (PMC12632903; doi:10.1101/2025.10.12.681832)
Supplement: 2 [file NIHPP2025.10.12.681832v2-supplement-2.pdf]

bioRxiv preprint doi: https://doi.org/10.1101/2025.10.12.681832; this version posted October 27, 2025. The copyright holder for this preprint (which was not certified by peer review) is the author/funder, who has granted bioRxiv a license to display the preprint in perpetuity. It is made available under aCC-BY 4.0 International license.

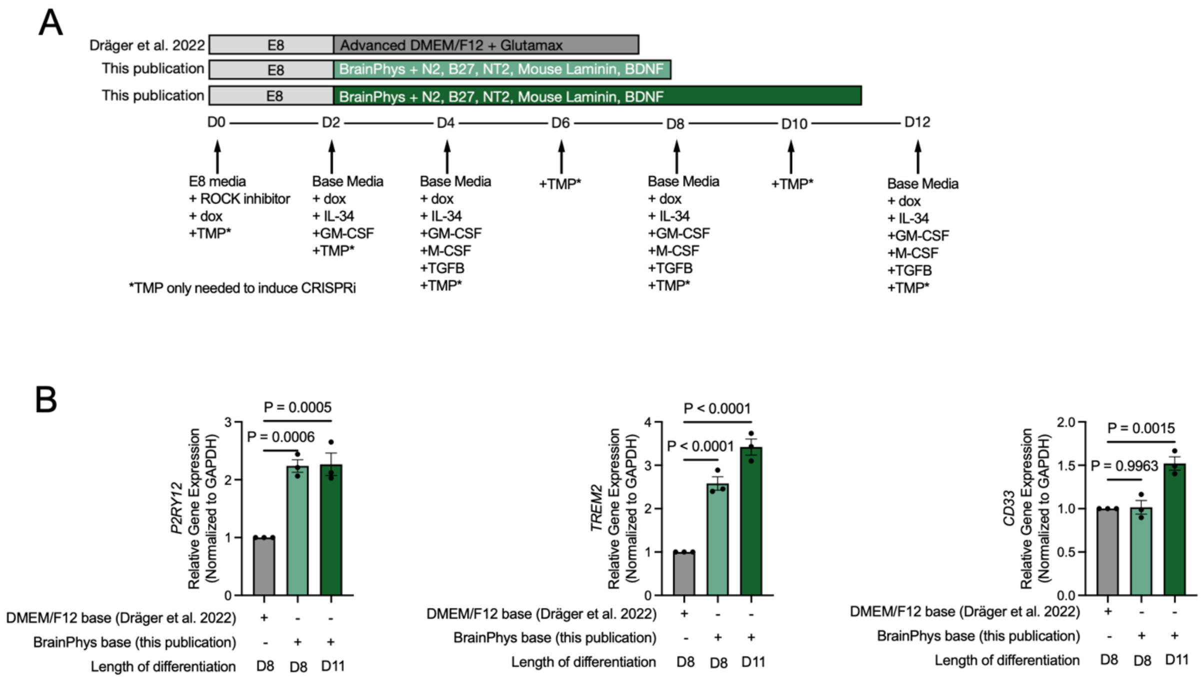

Figure S1. Updated differentiation protocol increases expression of homeostatic markers. (A) Schematic of the original and updated media composition and differentiation lengths. (B) Reverse-Transcription quantitative PCR analysis of homeostatic microglia markers (P2RY12, TREM2, CD33) in the original and updated media compositions. P value one-way ANOVA with Tukey post-hoc testing.

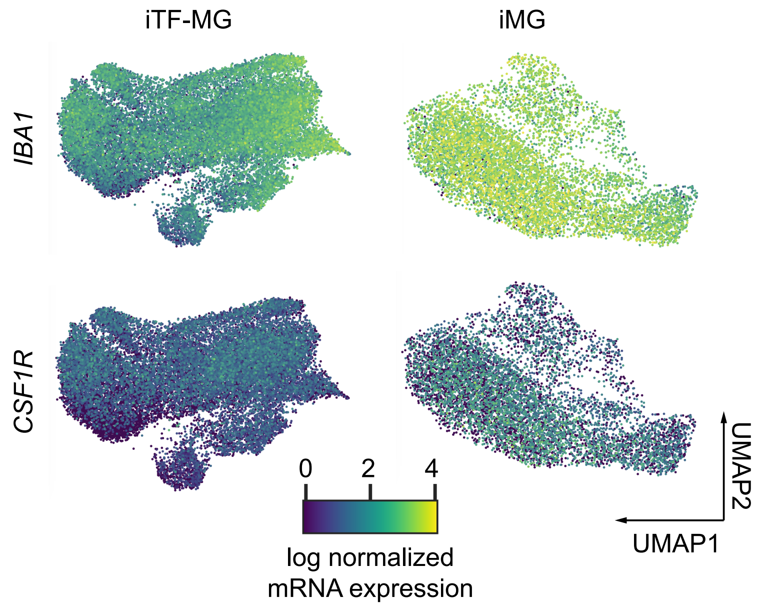

Figure S2. iTF-MG and iMG express microglia identity markers. UMAP representation of iTF-MG (left) and iMG (right) mRNA space. Cells are pseudocolored by expression levels of *IBA1* (top) and *CSF1R* (bottom).

bioRxiv preprint doi: https://doi.org/10.1101/2025.10.12.681832; this version posted October 27, 2025. The copyright holder for this preprint (which was not certified by peer review) is the author/funder, who has granted bioRxiv a license to display the preprint in perpetuity. It is made available under aCC-BY 4.0 International license.

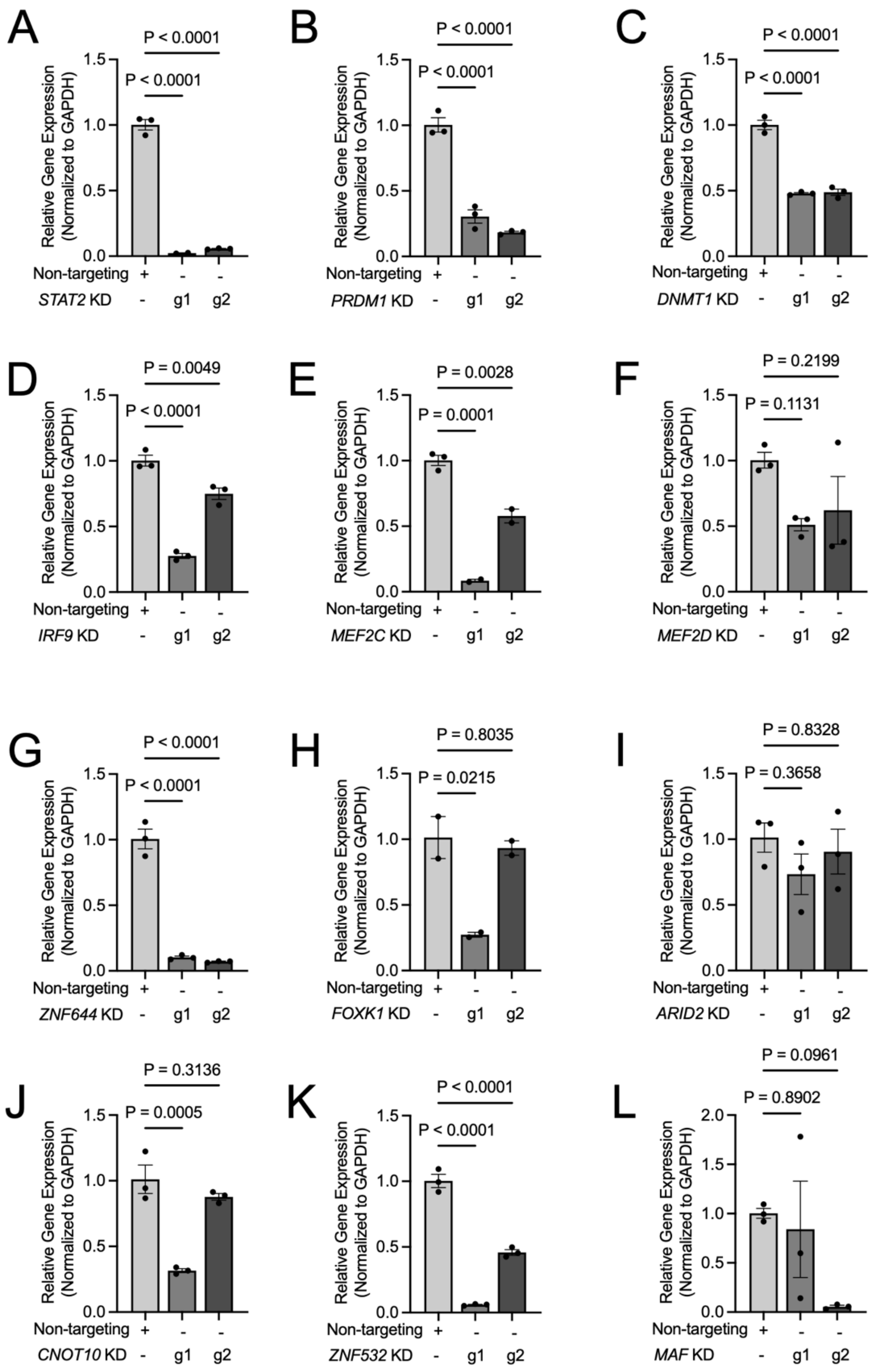

Figure S3. Gene expression knockdown validation for key sgRNAs. (A-L) Reverse-transcription quantitative PCR analysis of targeted genes after induction of CRISPRi. P value one-way ANOVA. *STAT2, DNMT1, IRF9, CNOT10* were performed in iTF-MG. *PRDM1, MEF2C, MEF2D, ZNF644, FOXK1, ARID2, ZNF532,* and *MAF* were quantified in iMG.

bioRxiv preprint doi: https://doi.org/10.1101/2025.10.12.681832; this version posted October 27, 2025. The copyright holder for this preprint (which was not certified by peer review) is the author/funder, who has granted bioRxiv a license to display the preprint in perpetuity. It is made available under aCC-BY 4.0 International license.

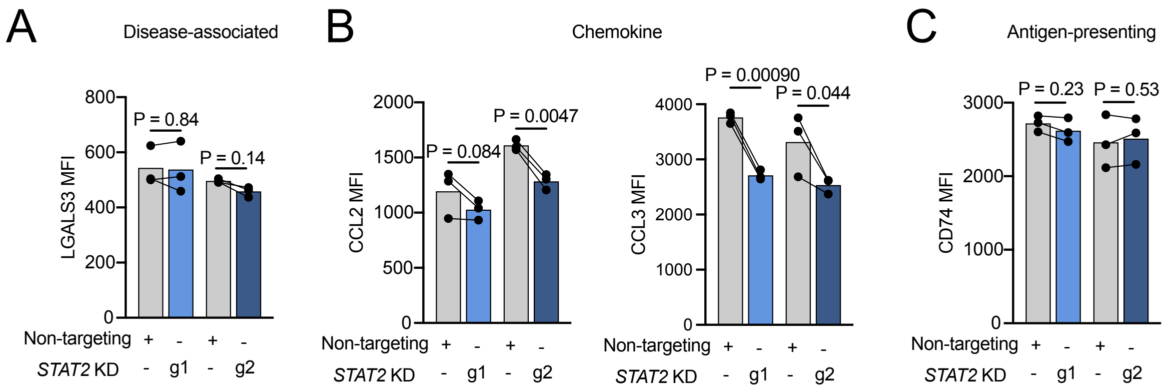

Figure S4. Additional quantification of protein state markers in *STAT2* KD. (A-C) Median fluorescence intensity (MFI) of state marker proteins by flow cytometry. *STAT2* KD microglia (blue) were compared to in-well non-targeting controls (grey) distinguished by nuclear fluorescent proteins. Points represent one well, n ≥ 10,000 cells analyzed per well. P value paired T-test.

bioRxiv preprint doi: https://doi.org/10.1101/2025.10.12.681832; this version posted October 27, 2025. The copyright holder for this preprint (which was not certified by peer review) is the author/funder, who has granted bioRxiv a license to display the preprint in perpetuity. It is made available under aCC-BY 4.0 International license.

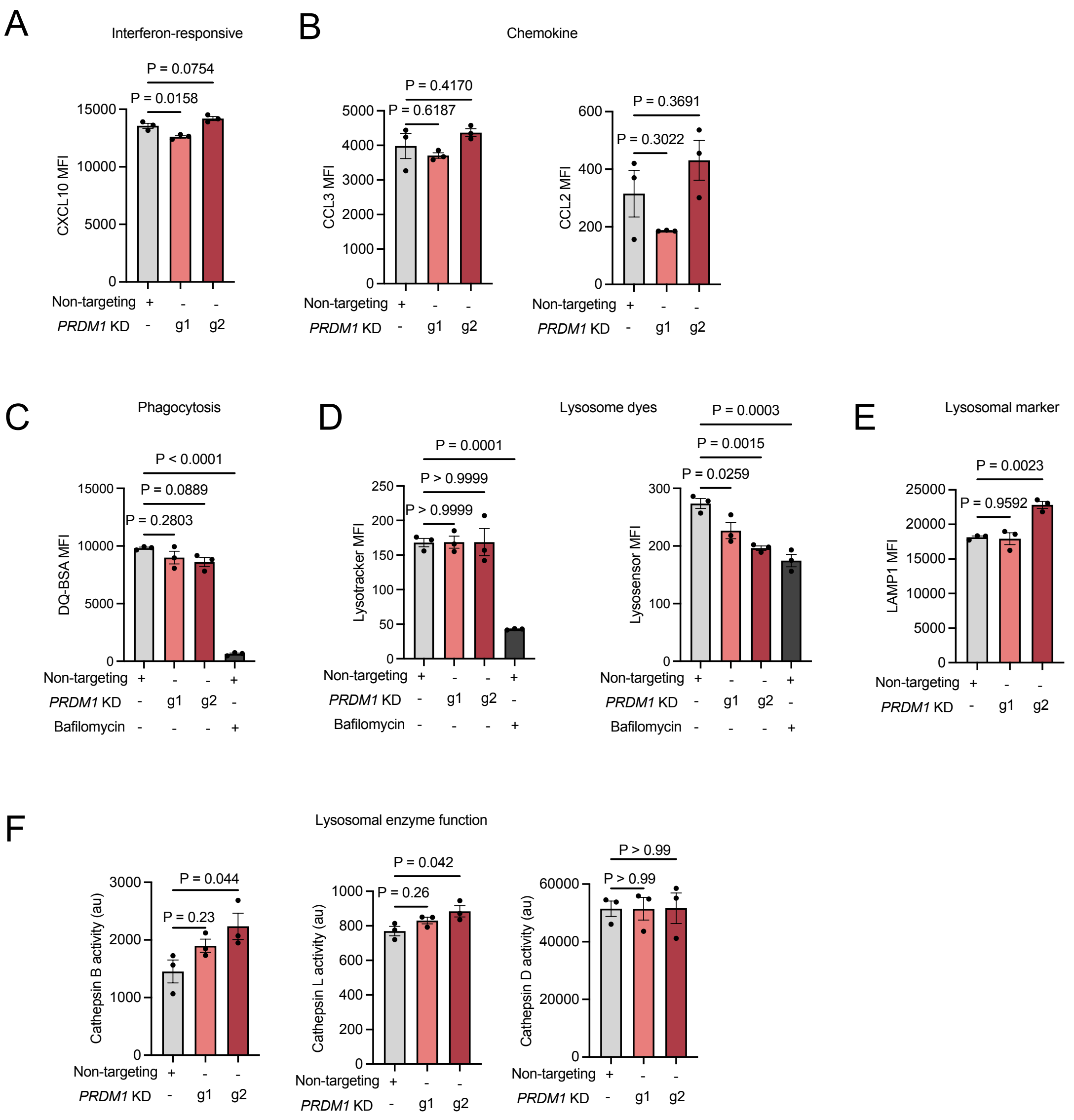

Figure S5. Additional quantification of protein state markers and lysosomal function in PRDM1 KD. (A-B) Median fluorescence intensity (MFI) of state marker proteins by flow cytometry. *PRDM1* KD microglia (salmon, red) were compared to non-targeting controls (grey). Points represent one well, n ≥ 10,000 cells analyzed per well. One way ANOVA. (C) Median fluorescence intensity (MFI) of DQ-BSA by flow cytometry. DQ-BSA was added for two hours before analysis to allow for uptake. Bafilomycin A was added four hours before analysis to control wells. Points represent one well, n ≥ 10,000 cells analyzed per well. One way ANOVA. (D) Median fluorescence intensity (MFI) of lysotracker and lysosensor in *PRDM1* KD microglia (salmon, red) and non-targeting controls (grey). Bafilomycin A was added four hours before analysis to control wells. Points represent one well, n ≥ 10,000 cells analyzed per well. One way ANOVA. (E) Median fluorescence intensity (MFI) of LAMP1 by flow cytometry. *PRDM1* KD microglia (salmon, red) were compared to non-targeting controls (grey). Points represent one well, n ≥ 10,000 cells analyzed per well. One way ANOVA. (F) Cathepsin activity measured in *PRDM1* KD microglia

bioRxiv preprint doi: https://doi.org/10.1101/2025.10.12.681832; this version posted October 27, 2025. The copyright holder for this preprint (which was not certified by peer review) is the author/funder, who has granted bioRxiv a license to display the preprint in perpetuity. It is made available under aCC-BY 4.0 International license.

(salmon, red) and non-targeting control cells (grey). Points represent independent wells, n ≥ 70,000 cells. One way ANOVA.

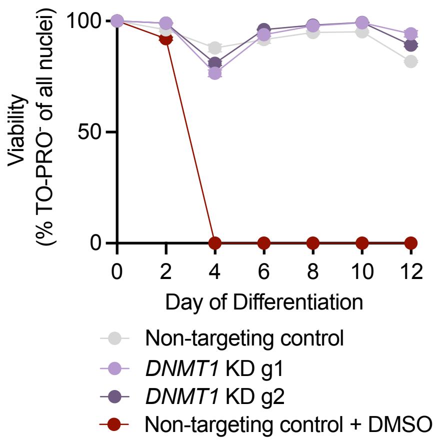

Figure S6. Knockdown of *DNMT1* does not alter viability. Viability of non-targeting control (grey) and *DNMT1* KD (violet) cells was measured every day of the differentiation into microglia by TO-PRO staining. n=2 wells, 4 images per well. 1% DMSO was added during normal media changes as a positive control for cell death.

bioRxiv preprint doi: https://doi.org/10.1101/2025.10.12.681832; this version posted October 27, 2025. The copyright holder for this preprint (which was not certified by peer review) is the author/funder, who has granted bioRxiv a license to display the preprint in perpetuity. It is made available under aCC-BY 4.0 International license.

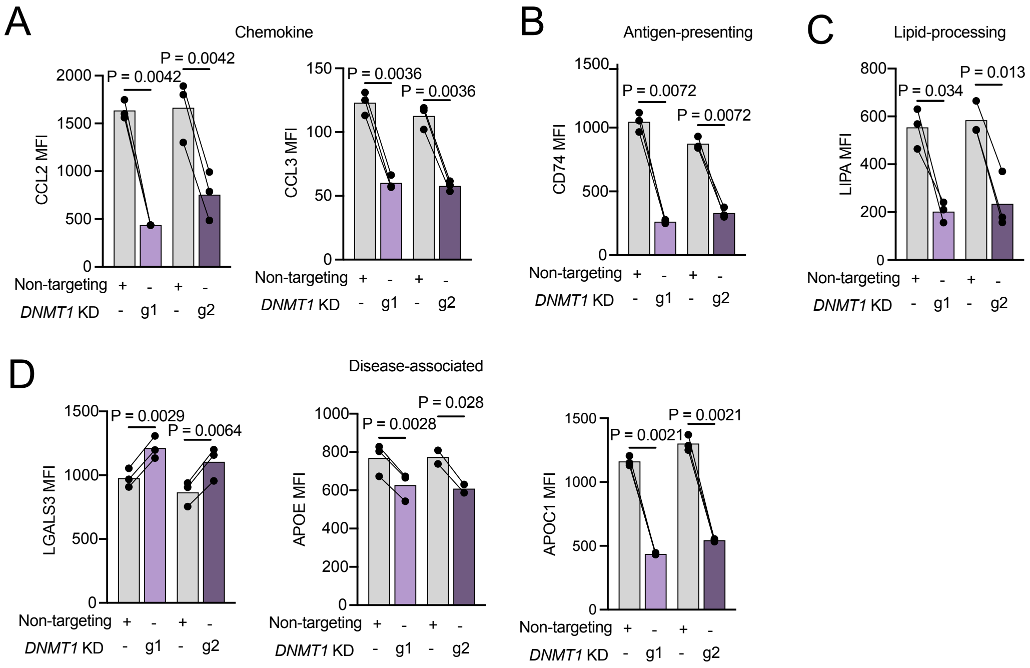

Figure S7. Additional quantification of protein state markers in *DNMT1* KD. (A-D) Median fluorescence intensity (MFI) of state marker proteins by flow cytometry. *DNMT1* KD microglia (violet) were compared to in-well non-targeting controls (grey) distinguished by nuclear fluorescent proteins. Points represent one well, n ≥ 10,000 cells analyzed per well. P value paired T-test.
